# A conservation planning strategy applied to the evolutionary history of the mantellid frogs of Madagascar

**DOI:** 10.1038/s44185-023-00024-4

**Published:** 2023-10-16

**Authors:** Miguel R. Ferreira, Francesco Belluardo, Walter Cocca, Angelica Crottini, Sílvia B. Carvalho

**Affiliations:** 1 CIBIO, Centro de Investigação em Biodiversidade e Recursos Genéticos, InBIO Laboratório Associado, Campus de Vairão, Universidade do Porto, 4485-661 Vairão, Portugal; 2 Departamento de Biologia, Faculdade de Ciências, Universidade do Porto, 4099-002 Porto, Portugal; 3 BIOPOLIS Program in Genomics, Biodiversity and Land Planning, CIBIO, Campus de Vairão, 4485-661 Vairão, Portugal

**Keywords:** Phylogenetics, Biodiversity, Conservation biology

## Abstract

Phylogenetic diversity is an increasingly applied metric used to maximize the representation of evolutionary history in spatial conservation planning. When following this approach, researchers commonly overlook sites with a relatively higher proportion of recently diverged endemic species, also known as centers of neo-endemism. Here we aim to demonstrate how targeting the conservation of different facets of diversity (taxonomic diversity, phylogenetic diversity and centers of endemism) can provide more cost-effective solutions to the conservation of the all evolutionary spectrum of biodiversity. We do so by using the mantellid frogs of Madagascar as a case study. Our results confirm that areas with high concentrations of neo-endemism can be effectively identified as conservation planning priorities only if we specifically target them. Neglecting areas that are poor in phylogenetic diversity may therefore compromise the maintenance of diversification processes, particularly when lesser proportions of the landscape are protected. This approach can be of particular interest to island ecosystems, since they often harbor unique and restricted evolutionary radiations.

## Introduction

More than ever before, we need protected areas (PAs) to halt the worrying declines of wildlife populations^1–3^. A slight expansion of land and sea surfaces committed to conservation can lead to immense benefits for biodiversity^4,5^ and the improvement of ecosystem services, human food provisioning, and health issues^6^. Hence, increasing the extension of PAs throughout the world remains a crucial point of global commitments such as the United Nations Sustainable Development Goals^7^ or the Convention on Biological Diversity^8^. One of the goals of the former Strategic Plan for Biodiversity Aichi Target 11 aimed at expanding the global network of PAs to cover 17% of the terrestrial landscape and 10% of the entire seascape by the end of 2020. The Kunming-Montreal Global Biodiversity Framework was recently ratified by 196 countries, who set more ambitious goals to protect at least 30% of the world’s lands, inland waters, coastal areas and oceans. However, there are several countries with high deforestation and land degradation rates, which do not have enough quality areas to convert to PAs and reach that goal. To maximize the chance of preventing additional biodiversity losses in these countries, it is crucial to ensure that existing PAs are effectively and equitably managed but also that they include the most relevant sites for conservation^9,10^.

When applied in practice, biodiversity conservation is a complex subject that may follow different approaches^11^. One of those is Systematic Conservation Planning (SCP), which provides parties with solid scientific frameworks that also comprise socioeconomic processes and policies^12–14^. This approach can identify the most suitable areas to be considered in plans for expanding PAs networks under pre-established conservation goals. Aiming at maximizing the long-term persistence of conservation features (usually species or habitats) through quantitative spatial prioritization methods, SCP considers key principles such as comprehensiveness, adequacy, representativeness, efficiency, and complementarity^15^.

Most SCP studies are based on species-level diversity^16,17^, but approaches addressing other facets of diversity, such as phenotypic traits and phylogenetic history, are increasingly common^18,19^. The idea that species are not equal in terms of the evolutionary history they embody resulted in considering phylogenetic relationships between species when prioritizing conservation efforts^20–22^. Following this concept, prioritization is undertaken under the assumption that areas capturing the maximum phylogenetic diversity (PD, Table 1) will also represent the highest diversity of evolutionary features. A popular approach in this field of research consists of maximizing the representation of PD in networks of PAs using SCP^23–25^.

**Table 1:** Indices of biodiversity richness and endemism addressed in this study.

Designation	Description
Species richness (SR)	Number of species occurring in a specific area.
Phylogenetic diversity (PD)	Amount of phylogenetic history represented by all species occurring in a specific area. It is calculated by the sum of the lengths of all phylogenetic tree branches involved from the root to the tips (each tip corresponding to a species).
Phylogenetic endemism (PE)	The uniqueness of phylogenetic history in a specific area. It is calculated by the sum of the phylogenetic tree branches of occurring species inversely weighted by their range size.
Relative phylogenetic endemism (RPE)	The ratio between two measures of phylogenetic endemism for a specific area: the phylogenetic endemism calculated using the original phylogenetic tree and the phylogenetic endemism calculated using an alternate phylogenetic tree where branch’s lengths were modified to have all the same length.
Centers of neo-endemism	Areas that concentrate a disproportionate amount of recently diversified species that are still confined to the region where they originated.
Centers of paleo-endemism	Areas that concentrate a disproportionate amount of anciently diversified species that suffered a considerable reduction in their geographical range and are presently confined to a small area.
Centers of mixed endemism	Areas that concentrate a disproportionate amount of both recent and ancient endemisms.
Centers of super-endemism	Centers of mixed endemism with an even higher level of significance.

See “Methods” section for more details.

An extension to the PD maximization method consists of identifying areas of phylogenetic endemism (PE, Table 1), for instance, by identifying centers of paleo-endemism, *sensu* Mishler et al.^26^. Centers of paleo-endemism are presumed to be formed by species that have been more widespread in the past and have contracted their range and are usually associated with current climatic seasonality and topographic heterogeneity, long-term geographical isolation, climatic uniqueness and stability, and higher energy availability^27,28^. The principle behind favoring the conservation of centers of paleo-endemism is that sets of species with more evolutionary history represent a higher proportion of the tree of life and capture more phenotypic and functional diversity^21^. However, the links between phylogenetic and functional diversity are not always clear^29^. The scientific community has been calling for more inclusive conservation measures of the evolutionary continuum and of the evolutionary potential. This is particularly relevant when taxonomy is uncertain^23,30^ and to allow species to develop adaptive responses to environmental disturbances^31,32^. The idea of protecting both centers of paleo- and neo-endemism (Table 1) has been proposed^26,33,34^.

Neo-endemic species are on the opposite spectrum of the evolutionary continuum, being species that originated relatively recently (i.e., have short phylogenetic branch lengths) and are generally confined to narrow ranges. They are presumed to be part of recently diverging clades, containing species that are endemic to the area due to the lack of dispersal/ migration out of their diversification area^26^. Mountains seem to play an important role in shaping spatial patterns of centers of neo-endemism, although contrasting factors have been found among different vertebrate groups^28^. Interestingly, some sites combine a set of both ancient and recent endemisms—centers of mixed endemism (Table 1)—or sites enclosing exceptional levels of endemism, the so-called centers of super-endemism^26^ (Table 1).

The identification of the sites that concentrate PE is a growing field of investigation, especially through the implementation of the categorical analysis of neo- and paleo-endemism (CANAPE)^26,28^. Different methodologies have been used, which have led to a clear conclusion that strengthens the theory behind CANAPE: centers of neo- and paleo-endemism tend to be fairly separated and frequently form cores of cells belonging to the same class in the geographical space regardless of the spatial resolution^34,35^, generally being found closer to centers of mixed endemism. Several recent methodological advances allow the integration of evolutionary data and processes into SCP (see refs. ^36,37^ for recent reviews). However, a methodology that allows the explicit integration of the different centers of endemism identified with CANAPE into a conservation planning framework is still missing.

Here we aim at demonstrating how targeting the conservation of different facets of diversity - taxonomic diversity, phylogenetic diversity, and centers of endemism can provide more cost-effective solutions to the conservation of the evolutionary spectrum of biodiversity. We do so by using the mantellid frogs of Madagascar as a case study.

Madagascar is one of the most celebrated biodiversity hotspots^38,39^, renowned for its exceptional number of endemic species^40^, most of which have evolved in isolation since the end of the Cretaceous period (ca 65 Mya)^39,41–47^ (Supplementary Note 1). Its biodiversity is unevenly distributed, with the majority of species located along the eastern rainforest belt and in the north^39^. Madagascar has been affected by massive rates of deforestation and forest degradation^44,48^, a threat that draws attention to its network of PAs (Supplementary Fig. S1), which has grown by more than a third over the last two decades^45^ (see also Fig. 3 in ref. ^10^).

Madagascar hosts exceptional levels of species diversity and endemism, particularly for amphibians. With 409 formally described species, at the time of writing (July 2023), (corresponding to 31% of all Malagasy vertebrate diversity), amphibians include several microendemic species, some resulting from the retraction of their geographical area due to past climatic events, while some others are only found in the areas where it has been hypothesized they have diversified. All amphibians of Madagascar are the result of 5 events of post-cretaceous colonization^39,42^. These had the opportunity to diversify within the island. For example, of the 409 formally described native amphibians, 267 (65%), species belong to the family Mantellidae^46^, which, except for three species (that are endemic to the Comoros), contains only species endemic to Madagascar. The mantellid frogs occur all over Madagascar, although their species richness (SR, Table 1) is unevenly distributed, being much higher in the Central East (Supplementary Note 2, Fig. S2,)^39^. Unlike of other vertebrate groups such as birds and mammals, the cataloging and description of the amphibian diversity of Madagascar are still far from being complete, with several lineages still requiring assessment and formal description (i.e. candidate species)^47,49^. Nonetheless, in comparison to other non-vertebrate taxonomic groups (e.g. invertebrates and fungi), the radiation of the mantellid frogs is relatively well known both in terms of species cataloging and species distributional data^39,50,51^. Therefore, we consider this lineage as an ideal case study to balance the conservation of paleo- and neo-endemisms and improve the alignment of conservation priorities for evolutionary radiations on islands.

To maximize the representation of the all evolutionary spectrum of biodiversity we followed a spatial conservation methodology where we used a prioritization algorithm to explicitly target both individual species distributions and centers of paleo- and neo-endemism (Scenario BrCE). We then compared results to the business-as-usual approaches where only taxonomic diversity is targeted through species distributions (scenario Tx), or both taxonomic and phylogenetic diversity are targeted (Scenario Br). We particularly focused on the proportion of centers of endemism covered by the different solutions, and assessed the extent to which the centers of paleo- and neo-endemism are already covered by the current network of protected areas. To do so, we revised the distribution of the mantellid frogs of Madagascar (including both formally described and candidate species) and described spatial patterns of species richness, phylogenetic diversity, phylogenetic endemism and centers of paleo-, neo-, mixed- and super-endemism.

## Results

### Spatial patterns of diversity and centers of endemism

**Fig. 1:**
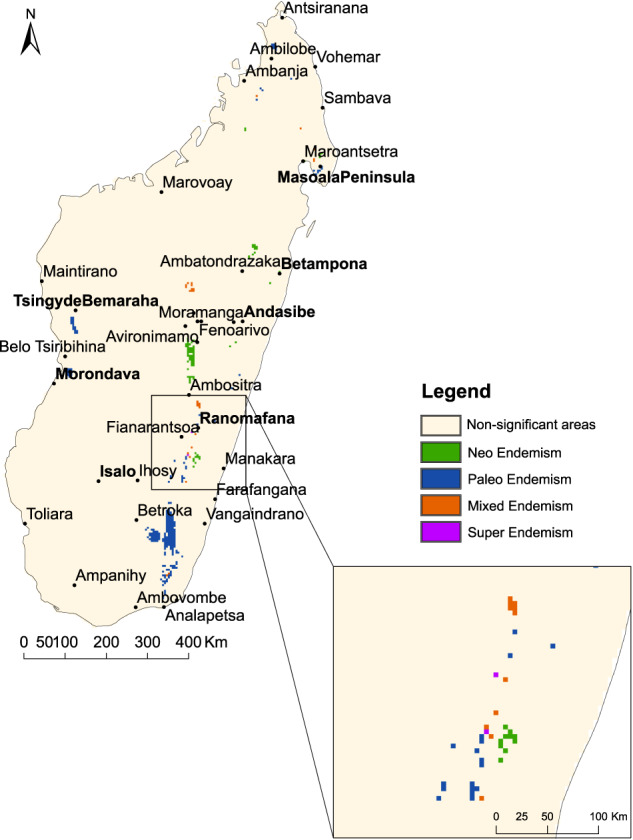
Centers of endemism for the mantellid frogs of Madagascar. Centers of neo-, paleo-, mixed- and super-endemism are displayed in different colors. The inset provides a zoomed view over the location of the two centers of super-endemism identified.

Spatial patterns of SR, PD and PE were unevenly distributed across Madagascar (Supplementary Fig. S2). In total, we identified 252 cells characterized as centers of paleo-endemism, 83 cells of centers of neo-endemism, 28 cells of centers of mixed endemism and 2 cells of centers of super-endemism (Fig. 1). The region southwest of Ranomafana was found to be the most diverse also in terms of categories of centers of endemism. The two super-endemic cells were identified in this area, along with paleo- and neo-endemisms. After Ranomafana, the Peninsula of Masoala was the area with the greatest diversity of categories, missing only the centers of super-endemism. Paleo-endemisms dominated in both the southeastern (south to Betroka) and western parts (south to Tsingy de Bemaraha) of Madagascar. Centers of paleo-endemism were identified (although at lower frequencies) also in northern Madagascar (around Ambilobe) and were rare in Central Madagascar (south to Avironimamo). Centers of neo- and mixed endemism were present mostly in the Central highlands and in Central East Madagascar, although a few cells were also recorded in the north.

### Spatial prioritization

**Fig. 2:**
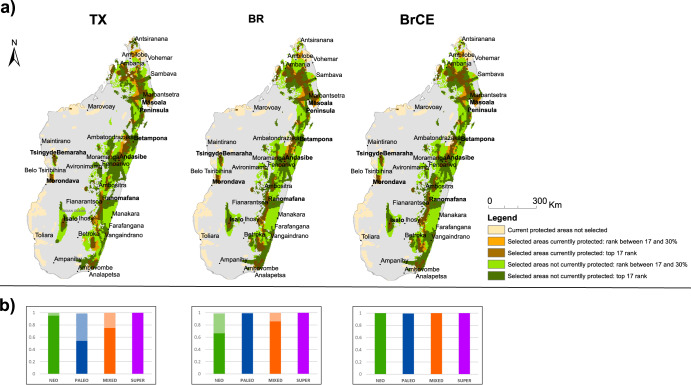
Spatial prioritization solutions and proportion of centers of endemism covered by the top 17% and 30% ranked cells. **a** Top 17% (dark green + dark brown) and the top 30% (all except gray and beige) selected cells in Zonation solutions for the different scenarios (Tx, Br and BrCE) and the fraction of the cells selected on the 17% and 30% top ranks that are included in current protected areas (orange and orange + brown, respectively); **b** Proportion of neo-, paleo-, mixed-and super- centers of endemism covered by the top 17% of zonation solutions (plain color pattern) and the extra amount covered by the 30% solutions (striped color pattern).

**Fig. 3:**
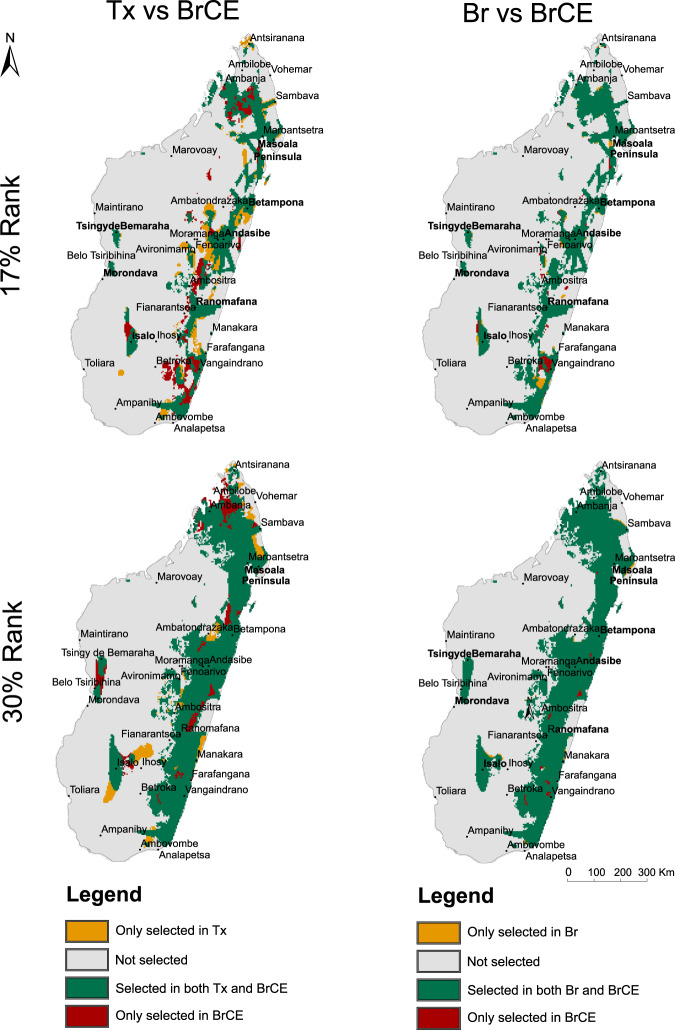
Spatial matches and mismatches of the selected cells in BrCE prioritization scenario, when compared to Tx (left column) and Br (right column) scenarios. The top row shows results for the top 17% ranked cells, while the bottom row shows results for the 30% ranked cells.

Eastern Madagascar was consistently selected as the major priority for the conservation of the mantellid frogs across all scenarios, although some western areas were also selected, namely the surroundings Menabe Antimena (near Morondava), Tsingy de Bemaraha and Isalo (Fig. 2, Supplementary Fig. S1). For the top 17% cells selected in the prioritization, Tx scenario (considering taxa distributions alone as conservation features; see the “Methods” section) unveiled a solution that overlapped both with Br (prioritization scenario targeting the lumped distribution of all species descending from each branch of the phylogenetic tree as conservation features) and with BrCE (prioritization scenario targeting the same conservation features as in Br scenario plus centers of endemism) in around 81.5% (4152 grid cells). A higher percentage was shared among Br and BrCE solutions: 96.6% (4917 grid cells), (see spatial matches and mismatches in Fig. 3). Considering the top 30% cells of the landscape, Tx matched in 92.0% (8271 grid cells) and 92.2% (8287 grid cells) of the solutions of Br and BrCE, respectively, while the two latter shared 99.2% (8920) of the selected grid cells (see spatial matches and mismatches in Fig. 3).

Despite this large overlap between solutions, we found relevant differences in terms of the centers of endemism selected by the prioritization solutions in the different scenarios, which were particularly accentuated in the top 17% rank of selected cells and lower percentages of selected landscape (Supplementary Fig. S3), while for the top 30% rank, almost all centers of endemism were covered by zonation solution found in the three scenarios (Fig. 3). However, for the percentage of protected landscape lower than 17%, the proportion of centers of endemism covered by the different solutions was more accentuated. For instance, for the Tx scenario, the proportion of neo-endemisms included in the prioritization solution was high at the 17% top rank (Fig. 3) but diminished considerably throughout the 17% top rank, while the proportion of paleo-endemisms included in the prioritization solution, diminished after the 30% rank (Supplementary Fig. S3). In the Br and BrCE scenarios, the centers of super-endemism were a top priority, being fully covered both in the 17% and 30% rank solutions (Fig. 3), and the proportion covered by the prioritization solution diminishing only at the top 5% rank and 2% rank, respectively (Supplementary Fig. S3). Neo-endemisms were entirely included in the solution by the 2% rank in the BrCE scenario, whereas in the Br scenario, there were accentuated reductions in coverage over the 25% top rank. As expected, the proportion of paleo-endemisms was overall better protected in the Br scenario than in the other two scenarios.

**Fig. 4:**
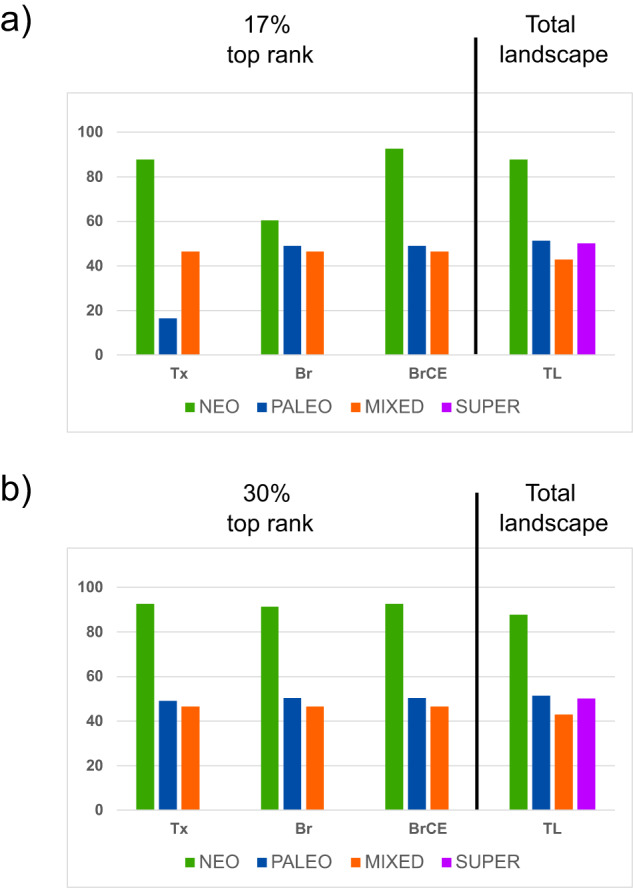
Percentage of grid cells of each type of center of endemism that are currently not covered by protected areas in the prioritization solutions found in each scenario (Tx, Br, BrCE). **a** Percentage of grid cells at the top 17% rank and **b** percentage of grid cells at the top 30% rank. The bars on the right side of the plots show the proportion of grid cells of each type of center of endemism that are covered by protected areas in the total landscape (TL). The different centers of endemism are color coded as in the legend, matching colors in Figs. 1 and 2.

Regarding the current protected area network, it only protects a fraction of identified centers of endemism: 12, 49, 57 and 50 percent of neo-, paleo-, mixed-, and super-centers of endemism, respectively. The percentage of centers of endemism selected in Zonation in the top 17% and 30% top ranks that are protected did not differ much between scenarios (Fig. 4). In the top 17% range selected in Zonation, the protected areas cover well the super-endemisms in all scenarios, but the non-protected cells complement particularly the neo-endemisms, especially in Tx and BrCE scenarios. In comparison with the proportion of centers of endemism currently protected in the total landscape (full study area), the solutions found in the prioritizations could improve the coverage of centers of endemism, particularly for mixed endemism and neo-endemism (the latter, particularly in the BrCE solution, found for the top 17% rank) (Fig. 4). In the top 30% range selected in Zonation, the additional cells selected in the zonation prioritization complement the coverage of centers of paleo-endemism in the Tx scenario and centers of mixed endemism in the Tx and Br scenarios.

All scenarios revealed a considerable improvement in the coverage of species distributions. We estimated that currently 291 (60.1%) mantellid frog lineages have less than half of their range included in PAs and 79 (16.3%) of them have less than 10% of their distribution within PAs. For the top 17% rank, the average percentage of range protection across the 484 considered mantellid species varied between 86.2% (Br) and 87.3% (Tx), almost doubling the current value (45.8%; Supplementary Tables S1.1–S1.4). In all scenarios, from the 79 species having less than 10% of their distribution currently included within PAs, only one remained in that situation.

## Discussion

Biodiversity patterns remain arduous to fully uncover, especially in tropical regions, where the number of species is often remarkably high, the formal description of this diversity is limited, and the low accessibility of these areas remains a major challenge. However, increasing efforts to document their richness and evolutionary history have been providing valuable information for aligning conservation priorities. Over the years, conservation biologists have been debating the advantages that can be achieved by maximizing PD within PAs, but the importance of formally protecting rapid diversification processes and their potential for future diversification has rarely been considered. The framework we introduce here confirms that areas with high concentrations of these diversification processes can be effectively identified as conservation planning priorities only if we specifically target them. Furthermore, it shows that it is possible to prioritize both long and short phylogenetic branches with only a limited increase in the total area as suggested using only PD: Br and BrCE scenarios differed solely in 14.6% (239 grid cells) of their selected areas. This allows for attaining a conservation solution that accounts for both these distinct but complementary descriptors of biological diversity.

Most centers of endemism are already within existing protected areas. This is no surprise since the majority of biodiversity expeditions in the country were conducted there and general patterns of species richness remain congruous to collection efforts^39^. Even so, some centers of endemism are located in unexpected places, as is the case of Central Highlands.

Although we decided to explicitly define as priorities only the centers of neo-, mixed- and super-endemism, centers of paleo-endemism were not neglected due to the inclusion of the phylogenetic tree branches as conservation features, and the inherent principles of complementarity and efficiency used in the Zonation algorithm. This suggests that long branches will be invariably considered, emphasizing the need to focus on shorter branches and thus targeting future diversification since the areas where they concentrate (i.e. centers of neo-endemism) are expected to be the most active in terms of the current evolutionary process^26^. Otherwise, we risk undermining these sites and include in our conservation planning solution a larger number of potentially evolutionary dead-ends^52,53^.

If we look at the case of Betampona Strict Nature Reserve (Fig. 1, Supplementary Note 1, Fig. S1), it is easy to understand the concept of neo-endemism. About a third (21) of all 59 species occurring in that center of endemism occur uniquely in this region, and several of the species found there are sympatric sister species (e.g. *Spinomantis* sp. 9 and *Spinomantis* sp. aff. aglavei; *Boophis* sp. 25 and *Boophis* sp. aff. *marojejenzis; Boophis roseipalmatus* and *Boophis madagascariensis*). Nevertheless, more complex patterns might be at the origin of this categorization. For example, the greatest concentration of centers of neo-endemism is found in the high-altitude sites of Central Madagascar (Fig. 1). One locally endemic species (*Mantidactylus* sp. 19) and two more widespread species (*M. brevipalmatus* and *M. alutus)* are present in every cell of that core area. Their relative proximity in the phylogenetic tree of *Mantidactylus* sp. 19 and *M. alutus* can partially explain this categorization, but the presence of the microendemic *Blommersia kely* in almost all those cells may also play a strong role in it, just as the absence of *Gephyromantis* and *Guibemantis* species, which are clades rich in evolutionary history that would excessively increase phylogenetic diversity. *Mantidactylus* sp. 19 and *B. kely* are both characterized by small terminal phylogenetic branches and share virtually the same range distribution, being perfect examples of neo-endemisms since they may be defined as an analogous species that recently diversified and are now occupying a specific confined niche. We find a very different situation in the north, where both *Tsingymantis antitra* and *Wakea madinika* are found, and in the southeast, where *Bohemantis microtympanum* is present. All these species have long phylogenetic branches (they are the only extant species of their genus) and have a small range distribution (especially *T. antitra* and *W. madinika*), which turns their occurrence cells into centers of paleo-endemism.

The identification of numerous paleo-endemic cells could be partially explained by the high diversification that took place in the early evolutionary history of this group, around 40–55 Mya^42,54^. This old diversification possibly played a key role in the adaptive radiation of this clade^55^, something which is reinforced by the current occurrence of similar (although not conspecific) ecomorphs in different localities^56^. Adaptive radiations are common in oceanic islands, resulting from the stochastic colonization of a limited number of colonizers and the wide exploitation of unoccupied niches^57^, a process which is in line with the biogeographical history of Madagascar, despite this being a large continental island^42,58,59^, although a recent study argues that the mantellid radiation shows insufficient diversification rates to be considered adaptive^60^.

We should acknowledge that certain methodological aspects may have influenced the results. For instance, species distributions were inferred mostly with minimum convex polygons, based on field records. This method is especially sensitive to sampling effort, which tends to be higher in Central-East Madagascar, particularly in protected areas and easily accessible areas, compared to areas in the west, north and southeast^39^ where more sampling efforts should be deployed. However, the database of species records compiled specifically for this study is almost triplicating the list of available mantellid species records used in previous studies (e.g. ref. ^50^) and is considered a robust proxy of the analyzed species distributions. Other methodological aspects are also worth mentioning, e.g. the removal of identified centers of endemism where only *Laliostoma labrosum* was present, and the removal of unsuitable grid cells (see Supplementary Fig. S4). If we had decided not to remove those grid cells, prioritization solutions would comprise a wide range of unsuitable areas for most mantellid frogs, diminishing their overall conservation value. Weighting species differently, based on their IUCN Red List classification, could be a way to avoid introducing such ad hoc manipulations. However, IUCN Red List assessments are often not updated and predominantly include described species. For example, at present, only 212 mantellid frogs of Madagascar (less than half of the lineages that we used here) have been evaluated by IUCN criteria^61^, rendering that alternative less effective. Another methodological choice worth mentioning was assuming that all grid cells had the same cost, for the sake of simplicity, and we did not account for any type of connectivity between selected sites. However, a more realistic cost estimation and the aim to maximize connectivity could have identified different trade-offs among scenarios. Finally, different sources of uncertainty in the datasets used could have influenced the results, including, for instance, uncertainty in phylogeny inference, which could have affected the tree topology and its branch lengths^62^.

Although endemism patterns have long been studied and mapped in Madagascar^39,63–65^, their relationship with PD is vastly unknown. As an exception, Isambert et al.^66^ have found that endemic beetle species resulting from recent radiations tend to occur in areas with low PD. Camacho et al. 2021^34^, studying acrobat ants, found low PD mostly in central and south-central Madagascar, and a concentration of neo-endemism at high elevations in the north and south-central Madagascar, while they found high PD at lower elevations along the East coast and the northwest of Madagascar and more palo-endemisms sites in the northwest. Our results somehow sit in an intermediate place, with most neo-endemism sites found at high altitudes in central eastern Madagascar, although several scattered neo-endemism sites are also found at lower altitudes along the eastern coast, whereas paleo-endemism are found at different altitudes in the northwest, west and southeast, while a high level of PD was found in the Central East and in the Northeast of Madagascar.

Amphibians are an iconic taxon in Madagascar and a better understanding of the spatial patterns of PD in Madagascar through the study of other taxa can reveal important conservation gaps. The present study has the goal to showcase an original approach to spatial planning, that also targets centers of diversification, rather than providing guidelines for ground implementation, which should be advocated only if a more comprehensive dataset (using multiple radiations) would have been used^17^. Although we relied on a single clade diversification pattern, we used an almost complete dataset for this radiation and complemented its use with the preparation of a revised distribution database for each analyzed lineage. In addition, it is worth noting that the mantellid radiation represents almost one-third of all vertebrate diversity in Madagascar^39,67^, and due to their widespread distribution across the island, it was possible to analyze Madagascar in most of its geographic extension. Finally, different vertebrates and plants clades have shown similar patterns of species richness and phylogenetic diversity^39^. We expect that similar results can potentially be obtained for other Malagasy biodiversity groups, especially those that show similar patterns of SR^68^ (with increased SR along eastern tropical forests^50,63,69,70^). Today, the once continuous rainforest belt is characterized by a multitude of relict forest fragments, most of them harboring disproportionate numbers of co-habiting species^71^, surrounded by deforested land. This fragmented landscape represents one of the major challenges for the effective conservation of Malagasy fauna and flora in the future^66,72^.

Rather than considering this work as a conservation recommendation, the current study is meant to serve as a showcase of the potential of weighting distinct aspects of the evolutionary history of a given group. Different outcomes will be obtained using different taxa, different measures of diversity, or different socio-political or economic constraints. To translate this approach into conservation guidelines, a broader taxonomic coverage should be considered and improving the effectiveness of existing protected areas (while tackling the main causes of biodiversity loss such as poverty and food security) may be more important than creating new ones^10^.

At present, these areas include the highest quality habitats in Madagascar and represent a huge opportunity for the conservation of the mantellid frogs, which have 93 species (43.5% of the evaluated lineages) currently threatened with extinction^61^. Assuring effective management of current PAs, (through the maximization of both their sustainable use and the well-being of Malagasy communities), the promotion of habitat protection and restoration, and the expansion of its network following rigorous scientific and technical criteria can be the way to ensure the long-term persistence of the biodiversity of Madagascar. Systematic Conservation Planning is an efficient way to accomplish some of the ambitious targets recently set by the international community in the context of the ongoing biodiversity crisis. Approaches such as the one described here provide valuable information to achieve representative sets of PAs, not only in terms of the number of taxa and PD, but also prioritizing microendemisms and places concentrating taxa that are diversifying and embody an enormous evolutionary potential.

## Methods

### Species list and distribution data

We compiled a database with distribution records for 493 mantellid lineages of Madagascar (including 267 formally described species and 226 candidate species, hereafter all referred to as species) whose phylogenetic relationships have recently been investigated^73,74^ (Supplementary Table S2, Figure S5, Data S1). Nine species have not been included in this phylogenetic hypothesis (either because samples for molecular analyses were not available: *Spinomantis brunae*, *Spinomantis nussbaumi*; or because their distribution records were too imprecise: *Blommersia* sp. aff. *blommersae*, *Boophis* sp. aff. *entigae* Bealanana, *Boophis* sp. aff. *elenae*, *Guibemantis* sp. aff. *liber* Makira 1, *Gephyromantis* sp. Masoala, *Mantidactylus* sp. CaNEW Makay and *Mantidactylus* sp. Ca66) and were therefore removed from our spatial analyses, which considered the remaining 484 species (see Supplementary Table S2). Two mantellid frog species, which are endemic to the Comoros, (*Blommersia transmarina* and *Boophis nauticus*), and two outgroups (*Polypedates* sp. and *Heterixalus variabilis*) were used to reconstruct the phylogenetic hypothesis (Supplementary Fig. S5, Data S1) but were removed from the topology used in this study.

We revised, compiled and merged two types of distribution data: (i) genetically confirmed occurrences, obtained from Genbank or unpublished sequences; and (ii) occurrences obtained from field guides^67,75,76^, institutional catalogs (mostly the catalog of the Museo Regionale di Scienze Naturali di Torino, Italy; and the catalog of the Zoologische Staatssammlung, München, Germany) and previously published datasets^50^. When 16 S rRNA sequences were unavailable, we assigned the taxonomic identification following an expert-based criterion, retrieving information on morphological characters. When an occurrence data could not be assigned with certainty to a species, it was removed from the database. We referenced all presence records of the World Geodetic System 1984 (WGS 84) and systematized them in a squared grid with 29929 cells of 2.5 arcminutes size (approximately 4.6 km × 4.6 km) using ArcGIS Desktop v10.5^76^.

### Species distribution ranges

To infer the distribution of each species, we estimated a coarse range based on the occurrence records and subsequently filtered out areas based on expert knowledge (removal of unsuitable elevation ranges and land uses). Depending on the total number of species records and their spatial clustering, we followed different approaches to estimate the coarse range. For species with at least two records, we computed Minimum Convex Polygons (MCPs) using the ‘*raster*’ R package. For species with more than three records and known to be continuously distributed, we generated a unique MCP with all records. Otherwise, we aggregated the available presence records in different spatial clusters (each cluster consisted of points that were geographically close to each other and distant from points forming other clusters) and calculated an individual MCP for each of them. For species with two occurrence records, we created a buffer of 4.6 km width (equal to grid cell size to account for spatial uncertainty) for each record, generated 100 random points within those buffers, and used these points to produce the MCP. For species that can be unambiguously diagnosed in the field and lacked considerable geographical information in our dataset (*Aglyptodactylus inguinalis*, *Blommersia blommersae*, *Mantella manery*, *Boophis tasymena*, *Boophis quasiboehmei*, *Boophis periegetes*, *Boophis guibei* and *L. labrosum*), we complemented the information of our MCPs with geographical information available in the IUCN Red List of Threatened Species website^61^. For species with one single record, and all the MCPs computed for species with more than one record, we generated a buffer with a width equal to the grid cell size. These areas (MCPs plus buffers) were considered the species’ coarse range. Overall, the species with more than one MCP were the more range-restricted and habitat-specialist species, and the ones with large geographical gaps between groups of records.

To bring these estimations of range distributions closer to reality, we filtered out unsuitable locations from the coarse range. We converted each coarse range to a raster format, using the R package ‘*raster*’^77^, and contrasted it with three layers: (i) the elevation^78^ and the cells of the landscape dominated by (ii) croplands or (iii) urban areas. For each species, we calculated the effective elevation range based on presence records and removed grid cells with elevation values found above or below that range. We also excluded all grid cells dominated by croplands, identified using a Land Cover map of Madagascar^79^, and urban areas, by manually drawing polygons around the biggest and most populated Malagasy cities in Google Earth v7.3.2.5776^80^ and posteriorly converting those polygons to a raster format. We did not exclude any cell of the MCPs that included at least one known presence record as available in the distribution database built specifically for this study.

### Spatial patterns of diversity and centers of endemism

We mapped SR, summing the number of species occurring in each grid cell, and employed the phylogeny^73^ (Supplementary Data S1) to obtain the spatial patterns of PD^21^ and PE^30^. Phylogenetic diversity was calculated by summing in each grid cell the overall branch lengths from the root of the phylogenetic tree to the tips of the occurring species using the R package ‘*picante*’^81^. Phylogenetic endemism was computed by summing the ratio between branch length and branch range for each branch using customized functions in R.

We employed the categorical analysis of neo- and paleo-endemism (CANAPE), a statistical method introduced by Mishler et al.^26^ that allows for discrimination between areas of endemism across space. In particular, it allows for explicitly identifying regions dominated by endemic species with long evolutionary histories—centers of paleo-endemism; and regions concentrating on recently diversified species—centers of neo-endemism. The CANAPE method, here executed in R through customized scripts, unfolded in 3 steps: (i) computation of relative phylogenetic endemism (RPE) (Table 1), which is the ratio between the observed PE and the same metric using an alternate phylogenetic tree. This alternate tree had the same topology as the original tree, but its branches were modified to have the same length (equal to the average branch length of the original tree); (ii) randomization of the matrix of species’ occurrences (i.e. the distribution of cells of each taxon were randomly selected from the landscape without replacement) while maintaining constant the number of species in each cell and the total number of occurrences of each taxon. This randomization was done 999 times and, for each iteration, the observed PE, the PE of the alternate tree and the RPE were calculated for each cell. Then, the significance of the observed PE was assessed by calculating its rank among the equivalent simulated values. This was done using a two-tailed test, which discriminates as significant the values that are ranked among the highest or lowest 2.5% (*α* = 0.05). In other words, all cells found to be significantly endemic either had higher or lower levels of PE than 95% of the 999 replications that were carried out; (iii) in the third step, each significant cell was classified into one of 4 categories: paleo-endemism, neo-endemism, mixed endemism or super-endemism. Paleo-endemisms and neo-endemisms corresponded to the cells whose RPE ratio was significantly high or low, respectively. Cells whose RPE ratio’s numerator and denominator were both significant, but the overall ratio was not, were considered mixed-endemisms. If these same conditions were verified but the RPE ratio’s numerator and denominator were both significant considering a threshold of *α* = 0.01, those cells were labeled as super-endemisms.

Most cropland and urban cells were removed from the coarse ranges of all species, which resulted in a null value of species richness and thus a null value of PD and PE. These cells were thus identified as centers of neo-endemism since their PE values ranked among the lowest 2.5% portion of the hypothesis test curve. However, since these cells are neither rich in species nor hotspots of recent diversification, we relabeled them as non-significant areas (see Supplementary Fig. S4). A similar methodological drawback happened with cells where only the species *L. labrosum* was present. This species is common in western Madagascar and given its phylogenetic distinctiveness (resulting in high PD, and consequently, high RPE), some cells where this species was the only one reported were classified as centers of paleo-endemism. These cells represent a vast area where all mantellid frogs are absent, except for the widespread species (*L. labrosum*). Considering that the goal of the present study is the prioritization of microendemic species, which require special attention due to the inherent vulnerability of their small and localized populations, we did not account for the phylogenetic distinctiveness of *L. labrosum*. As such, all cells where only *L. labrosum* is predicted to occur were relabeled as non-significant areas (see Supplementary Fig. 4).

### Spatial prioritization

We used the Zonation algorithm v4.0^82^ to rank grid cells according to their priority for the conservation of the mantellid frogs. This software follows the maximum coverage formulation, trying to retain in the landscape as many conservation features as possible while the meta-algorithm hierarchically decreases the percentage of protected landscape. Zonation does this by iteratively removing grid cells based on the marginal loss approach: at each iteration, it discards the cell that minimizes the decrease in conservation value of the remaining landscape, whereas the conservation value can be calculated according to different removal rules. We used the core-area Zonation removal rule, which, for each iteration, calculates the marginal loss of all cells in the remaining landscape, and removes the cell with the lowest marginal loss. The marginal loss of each cell is calculated by identifying the conservation feature with the highest proportion of its range remaining in the cell, multiplying that proportion by the weight of the feature, and dividing it by the cost of the cell. In this way, conservation priorities are given to species with narrower ranges and higher weights occurring in cells with lower costs^82^.

We produced 3 conservation scenarios, each with different conservation features: (i) Tx— taxa distributions; (ii) Br—branches distributions; and (iii) BrCE—branches distributions and centers of endemism. Tx scenario is the business-as-usual scenario, where the conservation features were individual species, which were all equally weighted (weight = 1.0). In the Br scenario, the conservation features were the branches of the phylogenetic tree, and the distribution of each feature was the lumped distribution of all descendant taxa from the respective branch. We set the weight of each branch equal to its length relative to the sum of all branches in the tree. This scenario has been used recently in some studies^23,83^. In the BrCE scenario, the conservation features were all branches and centers of neo-, mixed- and super-endemism (centers of paleo-endemism were excluded because these are characterized by a relatively high PD and thus were inherently prioritized by including the branches of the phylogenetic tree as conservation features). The weights of branches were set equal to the ones in the Br scenario, and the weights for centers of neo-, mixed- and super-endemism were set to 1.0. This scenario is the main innovation of this work, and we expect that prioritization found covers a higher proportion of all centers of endemism, particularly when the fraction of protected landscape is lower (as higher levels of protection will tend to be more similar to a random prioritization). Zonation produces a solution ranking the importance of each grid covering the study area. This overall prioritization can be analyzed at different percentage area thresholds. For this study, we analyzed zonation results at the 17% and 30% thresholds, which correspond to the areas required in international commitments (Aichi targets and Kumming-Montreal agreement, respectively). We analyzed the extent to which these two solutions cover the different centers of endemism.

We further analyzed how the 17% and 30% solution match with the current protected areas network. Note that information on protected areas was not included as input data in Zonation prioritizations, and was designated based on other taxonomic groups and conservation features other than solely mantellid frogs. To do so, we used spatial data of protected areas provided by Goodman et al.^84^ in polygon format, which was overlaid with a grid of 2.5 arcminutes. We computed the percentage of each raster grid cell covered by protected areas and considered as protected those cells with coverage equal or higher to 25%. We compared the areas selected from each solution within the current network of PAs in Madagascar to discriminate five classes: (1) current protected areas not selected; (2) selected areas currently protected, rank between 17% and 30%; (3) selected areas currently protected, top 17% rank; selected areas not currently protected: rank between 17% and 30%; and (4) selected areas not currently protected, top 17% rank. We calculated the percentage of grid cells within each class. Furthermore, we calculated the proportion of each center of endemism covered by the selected grid cells for different proportions of prioritized landscape (top 17% rank and rank between 17% and 30%), and the number of grid cells of each type of center of endemism in the top 17% rank and the rank between 17% and 30% that are currently protected or not currently protected.

### Supplementary information

Suplementary_Material_final

## Data Availability

A Supplementary Material document includes information on the Study area (Supplementary Fig. S1), the mantellid frog species list used in this study (Supplementary Table S2), an image of the used phylogenetic tree (Supplementary Fig. S5), the original phylogenetic tree used in this study (Supplementary Data S1). Individual specie’s distribution polygons (MCPs) used in this study will be made available upon request sent by email to the corresponding author (silviacarvalho@cibio.up.pt).

## Code availability

The software used in data analysis includes ArcGIS Desktop v10.5; R Core Team (2022). R: A language and environment for statistical computing with the following packages: *raster*,*dismo*, *rgdal*, *picante*, *ape*, *geiger*, *phangorn*, *gtools*, *phylotools* and *SDMTools*; Google Earth v7.3.2.5776 ; Zonation v4. Customized R scripts used will be made available upon a request sent by email to the corresponding author (silviacarvalho@cibio.up.pt).
