# Supplementary material for: A conservation planning strategy applied to the evolutionary history of the mantellid frogs of Madagascar
Source: NPJ Biodivers. 2023 Oct 16;2:21. doi: 10.1038/s44185-023-00024-4 (PMC11332064; doi:10.1038/s44185-023-00024-4)
Supplement: Supplementary file 1 — Suplementary_Material_final [file 44185_2023_24_MOESM1_ESM.docx]

**Supplementary Note 1**

Madagascar is the largest island of the African continent and one of the largest islands in the world (587,041 km^2^). Ranging from approximately 12º and 26º S in latitude and 43º to 50º E in longitude, it is located in the afro-tropic biogeographic realm. The complex topography of Madagascar is characterized by a major mountain range that roughly divides the country from North to South into a humid East and a drier West. The East is characterized by steep slopes, while the central highlands decrease gradually towards the West (Figure S1).

This island was once part of the supercontinent Gondwana, and around 170 million years ago (Mya), tectonic forces lead to the fragmentation of Gondwana into two main fragments, which were smoothly segregated over the geological eras^1^. Approximately 100 Mya, Madagascar was already separated from Africa and remained attached to India and the Seychelles, from which it also got separated around 90 – 80 Mya ^1,2^. Madagascar is now characterized by a great heterogeneity of ecoregions ^3^, and its long history of biogeographic isolation created the opportunity for the few oversea dispersers to radiate into the multitude of microendemic species that can currently be observed on the island ^4^.


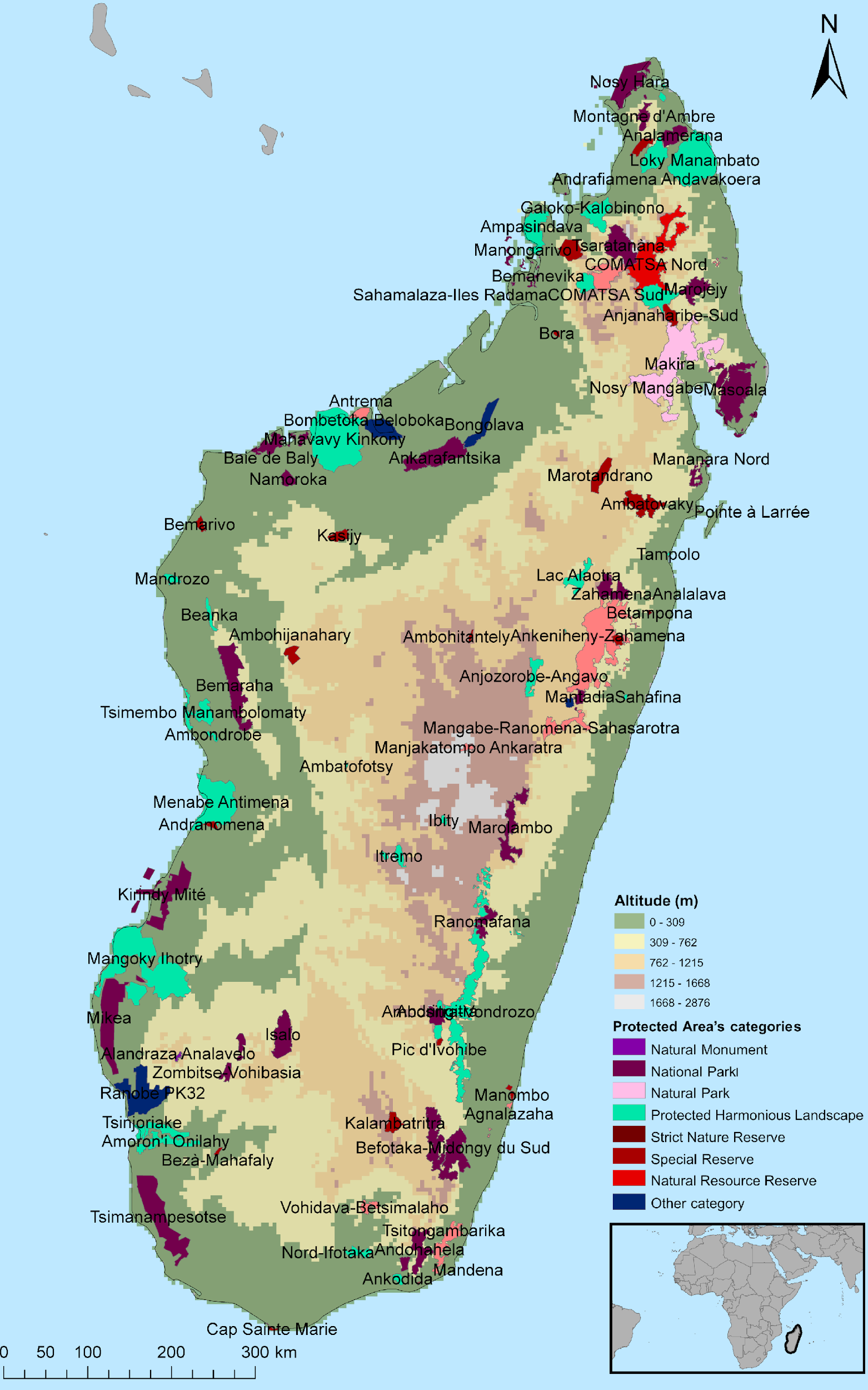


**Supplementary Figure S1 –** Study area (Madagascar), its elevation and current network of protected areas reviewed and edited according to Goodman, Raherilalao and Wohlhause ^5^ (including coastal protected areas, which were not included in our methodology). The inset shows Madagascar’s geographic location in the context of the African continent.

**Supplementary Note 2**

The eastern part of the country is characterised by disproportionate levels of SR, PD, PE (Figure S2). SR and PD displayed very similar patterns (Pearson correlation r^2^ = 0.9811), and we found their highest levels in Central East Madagascar, in the areas around and between Ranomafana and Andasibe. Northeast Madagascar was also highlighted by high levels of SR and PD. Spatial patterns of PE were also strongly correlated with those of SR (Pearson correlation r^2^ = 0.9395), peaking in a few restricted areas along the East and the North.

**
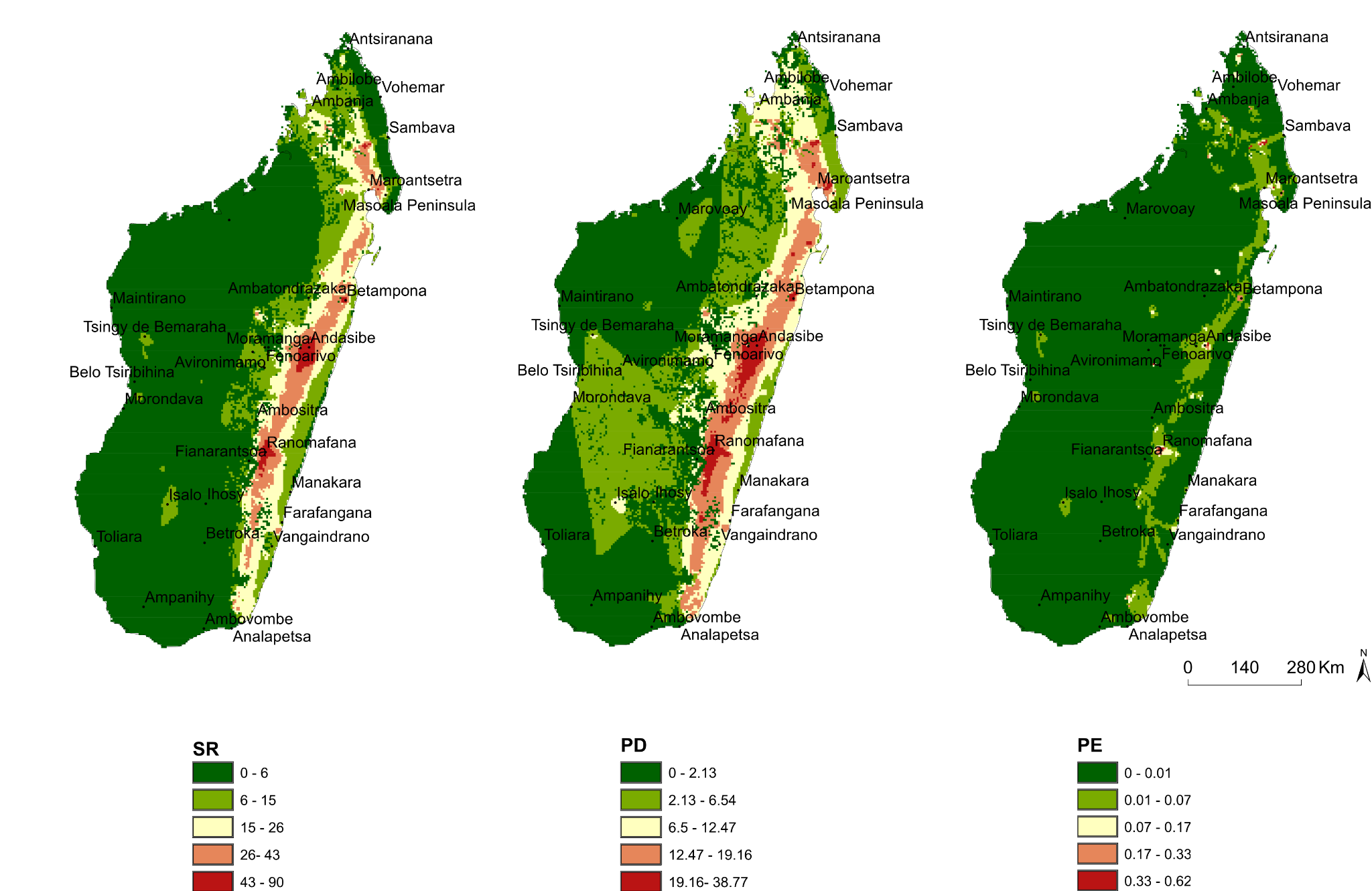
**

**Supplementary Figure S2 -** Spatial patterns of diversity for the mantellid frogs of Madagascar. Species Richness (SR), Phylogenetic Diversity (PD) and Phylogenetic Endemism (PE). Toponyms referred in the text are outlined.

**
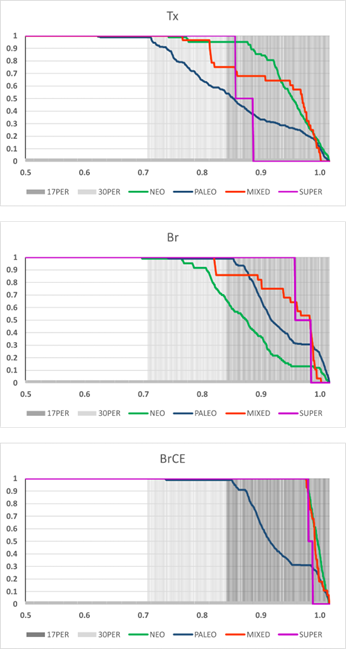
**

**Supplementary Figure S3** - Proportion of selected centres of endemism (y-axis) as the amount of unprotected land (x axis) increases. Each plot corresponds to a solution found by Zonation in the three different scenarios (Tx, Br and BrCE). The x-axis represents a different cut-off fraction of the number of grid cells removed, from half the landscape removed (0.5) to the whole landscape removed (1.0). The y-axis represents the fraction of each center of endemism remaining in the Zonation solution at the different thresholds. The different line colors represent the four types of centers of endemism, as depicted in the legend, with the same colour code as figure 12. The two shades of gray represent the 17% (dark grey) and 30% (light grey) thresholds, which were the ones represented in figure 1.

**Supplementary Table S1.1 –** Number of species included in each category of range coverage by current protected areas, and average protected range for each genus and for all mantellid species.

| Taxa | <10% | 10-25% | 25-50% | 50-70% | 70-80% | 80-90% | >=90% | Average protected range (%) |
| --- | --- | --- | --- | --- | --- | --- | --- | --- |
| *Aglyptodactylus* | 1 | 2 | 2 | 0 | 1 | 1 | 0 | 39.0 |
| *Blommersia* | 9 | 3 | 9 | 0 | 0 | 1 | 1 | 26.6 |
| *Boehmantis* | 0 | 0 | 1 | 0 | 0 | 0 | 0 | 39.9 |
| *Boophis* | 17 | 20 | 38 | 25 | 5 | 12 | 23 | 49.3 |
| *Gephyromantis* | 17 | 10 | 21 | 14 | 7 | 7 | 18 | 50.8 |
| *Guibemantis* | 9 | 9 | 15 | 8 | 0 | 1 | 7 | 40.0 |
| *Laliostoma* | 0 | 1 | 0 | 0 | 0 | 0 | 0 | 14.6 |
| *Mantella* | 4 | 4 | 11 | 0 | 1 | 0 | 0 | 27.6 |
| *Mantidactylus* | 21 | 22 | 34 | 17 | 2 | 5 | 19 | 43.5 |
| *Spinomantis* | 1 | 1 | 9 | 7 | 0 | 1 | 8 | 60.0 |
| *Tsingymantis* | 0 | 0 | 0 | 0 | 1 | 0 | 0 | 77.8 |
| *Wakea* | 0 | 0 | 0 | 0 | 0 | 0 | 1 | 100 |
| Total | 79 | 72 | 140 | 71 | 17 | 28 | 77 | 45.8 |

**Supplementary Table S1.2 –** Number of species included in each category of range coverage by the top 17% rank cells of the Tx scenario, and average protected range for each genus and for all mantellid species.

| Taxa | <10% | 10-25% | 25-50% | 50-70% | 70-80% | 80-90% | >=90% | Average protected range (%) |
| --- | --- | --- | --- | --- | --- | --- | --- | --- |
| *Aglyptodactylus* | 0 | 0 | 1 | 2 | 0 | 1 | 3 | 76.9 |
| *Blommersia* | 0 | 1 | 1 | 5 | 0 | 1 | 15 | 83.5 |
| *Boehmantis* | 0 | 0 | 0 | 0 | 0 | 1 | 0 | 82.0 |
| *Boophis* | 0 | 1 | 13 | 22 | 6 | 3 | 95 | 86.4 |
| *Gephyromantis* | 0 | 0 | 1 | 8 | 6 | 3 | 76 | 93.5 |
| *Guibemantis* | 0 | 0 | 1 | 10 | 6 | 2 | 30 | 85.8 |
| *Laliostoma* | 0 | 1 | 0 | 0 | 0 | 0 | 0 | 12.7 |
| *Mantella* | 1 | 0 | 3 | 4 | 2 | 2 | 8 | 74.7 |
| *Mantidactylus* | 0 | 0 | 9 | 20 | 5 | 5 | 81 | 87.3 |
| *Spinomantis* | 0 | 0 | 0 | 4 | 3 | 1 | 19 | 90.8 |
| *Tsingymantis* | 0 | 0 | 0 | 0 | 0 | 0 | 1 | 100 |
| *Wakea* | 0 | 0 | 0 | 0 | 0 | 0 | 1 | 100 |
| Total | 1 | 3 | 29 | 75 | 28 | 19 | 329 | 87.3 |

**Supplementary Table S1.3 –** Number of species included in each category of range coverage by the top 17% rank cells of the Br scenario, and average protected range for each genus and for all mantellid species.

| Taxa | <10% | 10-25% | 25-50% | 50-70% | 70-80% | 80-90% | >=90% | Average protected range (%) |
| --- | --- | --- | --- | --- | --- | --- | --- | --- |
| *Aglyptodactylus* | 0 | 0 | 2 | 1 | 0 | 0 | 4 | 78.5 |
| *Blommersia* | 0 | 1 | 2 | 5 | 1 | 2 | 12 | 80.0 |
| *Boehmantis* | 0 | 0 | 0 | 0 | 0 | 0 | 1 | 100 |
| *Boophis* | 0 | 1 | 17 | 14 | 10 | 1 | 97 | 86.2 |
| *Gephyromantis* | 0 | 0 | 1 | 12 | 4 | 7 | 70 | 91.7 |
| *Guibemantis* | 0 | 0 | 2 | 10 | 4 | 1 | 32 | 86.0 |
| *Laliostoma* | 0 | 1 | 0 | 0 | 0 | 0 | 0 | 13.2 |
| *Mantella* | 1 | 1 | 5 | 6 | 2 | 2 | 3 | 62.0 |
| *Mantidactylus* | 0 | 0 | 10 | 20 | 7 | 3 | 80 | 86.4 |
| *Spinomantis* | 0 | 0 | 0 | 3 | 1 | 1 | 22 | 93.6 |
| *Tsingymantis* | 0 | 0 | 0 | 0 | 0 | 0 | 1 | 100 |
| *Wakea* | 0 | 0 | 0 | 0 | 0 | 0 | 1 | 100 |
| Total | 1 | 4 | 39 | 71 | 29 | 17 | 323 | 86.2 |

**Supplementary Table S1.4 –** Number of species included in each category of range coverage by the top 17% rank cells of the BrCE scenario, and average protected range for each genus and for all mantellid species.

| Taxa | <10% | 10-25% | 25-50% | 50-70% | 70-80% | 80-90% | >=90% | Average protected range (%) |
| --- | --- | --- | --- | --- | --- | --- | --- | --- |
| *Aglyptodactylus* | 0 | 0 | 2 | 1 | 0 | 0 | 4 | 78.1 |
| *Blommersia* | 0 | 1 | 2 | 5 | 1 | 1 | 13 | 80.0 |
| *Boehmantis* | 0 | 0 | 0 | 0 | 0 | 0 | 1 | 100 |
| *Boophis* | 0 | 1 | 15 | 15 | 11 | 2 | 96 | 86.2 |
| *Gephyromantis* | 0 | 0 | 2 | 11 | 5 | 6 | 70 | 91.6 |
| *Guibemantis* | 0 | 0 | 2 | 11 | 3 | 2 | 31 | 85.8 |
| *Laliostoma* | 0 | 1 | 0 | 0 | 0 | 0 | 0 | 13.1 |
| *Mantella* | 1 | 1 | 4 | 7 | 1 | 1 | 5 | 63.4 |
| *Mantidactylus* | 0 | 0 | 10 | 20 | 6 | 3 | 81 | 86.5 |
| *Spinomantis* | 0 | 0 | 0 | 3 | 1 | 1 | 22 | 93.6 |
| *Tsingymantis* | 0 | 0 | 0 | 0 | 0 | 0 | 1 | 100 |
| *Wakea* | 0 | 0 | 0 | 0 | 0 | 0 | 1 | 100 |
| Total | 1 | 4 | 37 | 73 | 28 | 16 | 325 | 86.3 |


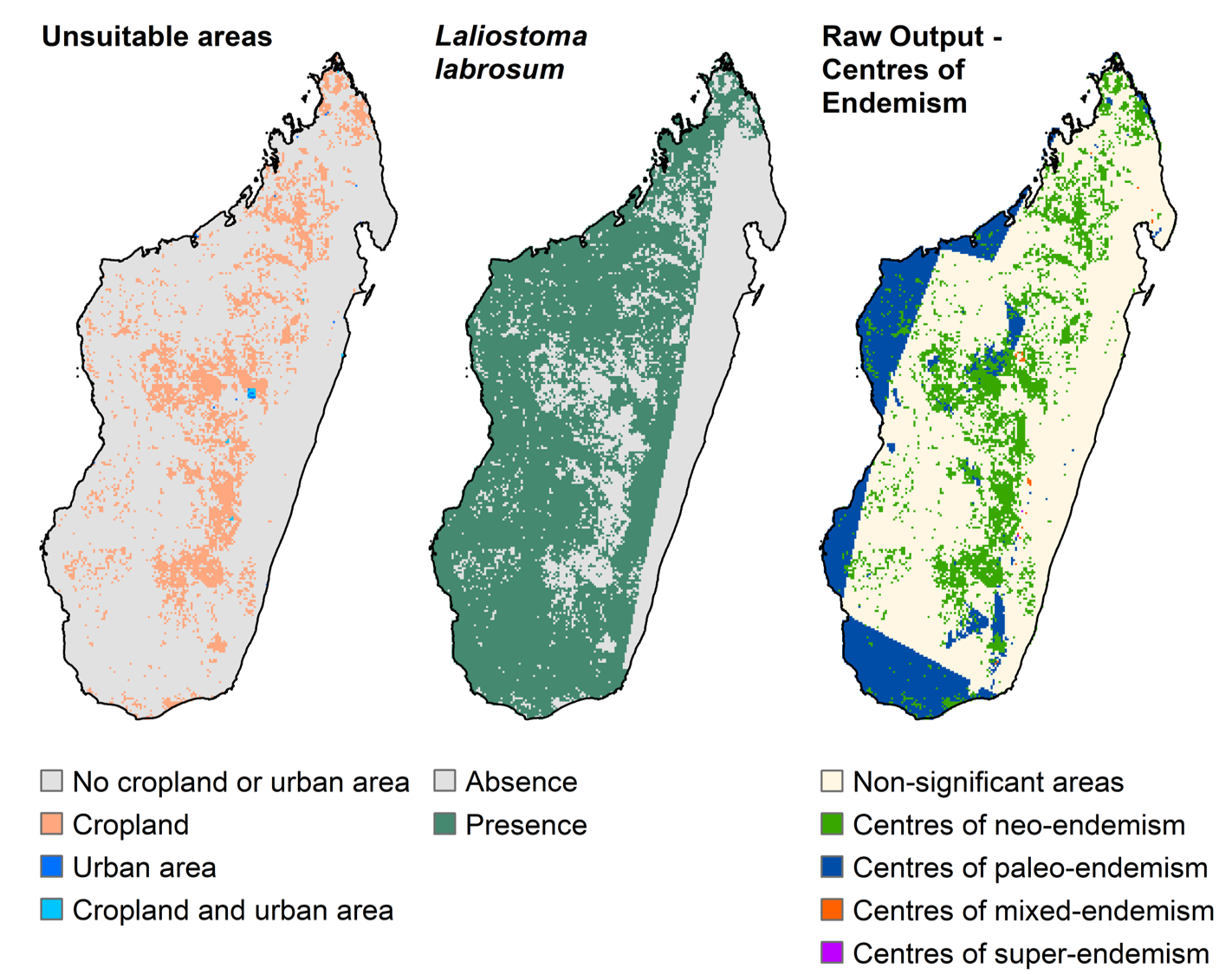


**Supplementary Figure S4 –** Cells occupied by cropland ^7^ and urban areas, the inferred distribution of the widespread species *Laliostoma labrosum*, the only species inferred to be present in most cells of western Madagascar, and the raw output of CANAPE ^8^. The influence of cropping cropland and urban cells from our methodology in the identification of centres of neo-endemism, and the influence of removing cells containing only *L. labrosum* in the identification of centres of paleo-endemism led to a post-processing step (see Methods’ section) to obtain the final layer of centres of endemism (figure 2).

**Supplementary Table S2 –** List of formally described and candidate species of mantellid frogs considered in this study (each with a unique ID number). The species that were excluded from spatial analyses due to their omission from the phylogeny (Supplementary Material 7; see Methods’ section for further details) are also listed.

| **ID** | **Species** | **Status** | **Used in spatial analyses** |
| --- | --- | --- | --- |
| 1 | *Aglyptodactylus inguinalis* | Described | Yes |
| 2 | *Aglyptodactylus chorus* | Described | Yes |
| 3 | *Aglyptodactylus laticeps* | Described | Yes |
| 4 | *Aglyptodactylus australis* | Described | Yes |
| 5 | *Aglyptodactylus madagascariensis* | Described | Yes |
| 6 | *Aglyptodactylus securifer* | Described | Yes |
| 7 | *Blommersia* sp*.* 9 | Candidate | Yes |
| 8 | *Blommersia kely* | Described | Yes |
| 9 | *Blommersia sarotra* | Described | Yes |
| 10 | *Blommersia* sp. 8 | Candidate | Yes |
| 11 | *Blommersia angolafa* | Described | Yes |
| 12 | *Blommersia grandisonae* | Described | Yes |
| 13 | *Blommersia* sp. 6 | Candidate | Yes |
| 14 | *Blommersia* sp. 13 | Candidate | Yes |
| 15 | *Blommersia* sp. 10 | Candidate | Yes |
| 16 | *Blommersia* sp. 11 | Candidate | Yes |
| 17 | *Blommersia* sp*.* aff*. blommersae* | Candidate | No |
| 18 | *Blommersia* sp. 5 | Candidate | Yes |
| 19 | *Blommersia wittei* | Described | Yes |
| 20 | *Blommersia blommersae* | Described | Yes |
| 21 | *Blommersia* sp. aff. *blommersae* "Ranomafana" | Candidate | Yes |
| 22 | *Blommersia domerguei* | Described | Yes |
| 23 | *Blommersia variabilis* | Described | Yes |
| 24 | *Blommersia galani* | Described | Yes |
| 25 | *Blommersia* sp. aff*. blommersae* "Ivoloina**"** | Candidate | Yes |
| 26 | *Blommersia dejongi* | Described | Yes |
| 27 | *Blommersia* sp. 12 | Candidate | Yes |
| 29 | *Blommersia* sp. CaNEW | Candidate | Yes |
| 30 | *Blommersia* sp. CaNEW | Candidate | Yes |
| 31 | *Blommersia* sp. CaNEW | Candidate | Yes |
| 32 | *Boehmantis microtympanum* | Described | Yes |
| 33 | *Boophis* sp. aff. *pauliani* "Tolagnaro" | Candidate | Yes |
| 34 | *Boophis* sp. aff*. pauliani* | Candidate | Yes |
| 35 | *Boophis pauliani* | Described | Yes |
| 36 | *Boophis tephraeomystax* | Described | Yes |
| 37 | *Boophis doulioti* | Described | Yes |
| 39 | *Boophis xerophilus* | Candidate | Yes |
| 40 | *Boophis* sp. aff. *guibei* | Candidate | Yes |
| 41 | *Boophis idae* | Described | Yes |
| 42 | *Boophis guibei* | Described | Yes |
| 43 | *Boophis* sp. CaNEW | Candidate | Yes |
| 44 | *Boophis calcaratus* | Described | Yes |
| 45 | *Boophis lichenoides* | Described | Yes |
| 46 | *Boophis opisthodon* 1 | Described | Yes |
| 47 | *Boophis opisthodon* 2 | Described | Yes |
| 48 | *Boophis tsilomaro* | Described | Yes |
| 49 | *Boophis albilabris* | Described | Yes |
| 50 | *Boophis praedictus* | Described | Yes |
| 51 | *Boophis occidentalis* | Described | Yes |
| 52 | *Boophis masoala* | Described | Yes |
| 53 | *Boophis* sp*.* 40 | Candidate | Yes |
| 54 | *Boophis* sp*.* 43 | Candidate | Yes |
| 55 | *Boophis* sp. aff. *fayi* | Candidate | Yes |
| 56 | *Boophis fayi* | Described | Yes |
| 57 | *Boophis quasiboehmei* | Described | Yes |
| 58 | *Boophis periegetes* | Described | Yes |
| 59 | *Boophis spinophis* | Described | Yes |
| 60 | *Boophis* sp. aff*. spinophis* "Ambatolahy" 2 | Candidate | Yes |
| 61 | *Boophis roseipalmatus* | Described | Yes |
| 62 | *Boophis madagascariensis* | Described | Yes |
| 63 | *Boophis* sp*.* 42 | Candidate | Yes |
| 64 | *Boophis goudotii* | Described | Yes |
| 65 | *Boophis obscurus* | Described | Yes |
| 66 | *Boophis brachychir* | Described | Yes |
| 67 | *Boophis entingae* | Described | Yes |
| 68 | *Boophis popi* | Described | Yes |
| 69 | *Boophis* sp. aff. *entingae* "Beanalana" | Candidate | No |
| 70 | *Boophis* sp. aff. *entingae* "Tsaratanana" | Candidate | Yes |
| 71 | *Boophis* sp. CaNEW | Candidate | Yes |
| 72 | *Boophis* sp. aff. *boehmei* | Candidate | Yes |
| 73 | *Boophis boehmei* | Described | Yes |
| 74 | *Boophis* sp. aff. *reticulatus* "Ranomafana" | Candidate | Yes |
| 75 | *Boophis reticulatus* | Described | Yes |
| 76 | *Boophis* sp. 41 | Candidate | Yes |
| 77 | *Boophis* sp. 7 | Candidate | Yes |
| 78 | *Boophis* sp. CaNEW | Candidate | Yes |
| 79 | *Boophis* sp. aff. *axelmeyeri* | Candidate | Yes |
| 80 | *Boophis axelmeyeri* | Described | Yes |
| 81 | *Boophis* sp. CaNEW | Candidate | Yes |
| 82 | *Boophis burgeri* | Described | Yes |
| 83 | *Boophis* sp. 10 | Candidate | Yes |
| 84 | *Boophis* sp. CaNEW | Candidate | Yes |
| 85 | *Boophis rufioculis* | Described | Yes |
| 87 | *Boophis solomaso* | Described | Yes |
| 88 | *Boophis* sp. 39 | Candidate | Yes |
| 89 | *Boophis* sp. 54 | Candidate | Yes |
| 90 | *Boophis* sp. 46 | Candidate | Yes |
| 91 | *Boophis* sp. 38 | Candidate | Yes |
| 92 | *Boophis* sp. aff. *solomaso* | Candidate | Yes |
| 93 | *Boophis sambirano* | Described | Yes |
| 94 | *Boophis* sp. 28 | Candidate | Yes |
| 95 | *Boophis* sp. Ca50 | Candidate | Yes |
| 96 | *Boophis* sp. 47 | Candidate | Yes |
| 97 | *Boophis* sp. 48 | Candidate | Yes |
| 98 | *Boophis* sp. Ca49 | Candidate | Yes |
| 99 | *Boophis* sp. aff. *mandraka* "Ranomafana" | Candidate | Yes |
| 100 | *Boophis mandraka* | Described | Yes |
| 101 | *Boophis* sp. 27 | Candidate | Yes |
| 102 | *Boophis liami* | Described | Yes |
| 103 | *Boophis blommersae* | Described | Yes |
| 104 | *Boophis* sp. aff. *blommersae* | Candidate | Yes |
| 105 | *Boophis vittatus* | Described | Yes |
| 106 | *Boophis* sp. aff. *vittatus* "Manongarivo" | Candidate | Yes |
| 107 | *Boophis* sp. Ca51 | Candidate | Yes |
| 108 | *Boophis* sp. 55 | Candidate | Yes |
| 109 | *Boophis* sp. Ca52 | Candidate | Yes |
| 110 | *Boophis* sp. 26 | Candidate | Yes |
| 111 | *Boophis* sp. 25 | Candidate | Yes |
| 112 | *Boophis marojezensis* | Described | Yes |
| 113 | *Boophis* sp. aff. *marojezensis* | Candidate | Yes |
| 115 | *Boophis albipunctatus* | Described | Yes |
| 116 | *Boophis sibilans* | Described | Yes |
| 117 | *Boophis luciae* | Described | Yes |
| 118 | *Boophis haingana* | Described | Yes |
| 119 | *Boophis boppa* | Described | Yes |
| 120 | *Boophis ankaratra* | Described | Yes |
| 121 | *Boophis schuboeae* | Described | Yes |
| 122 | *Boophis miadana* | Described | Yes |
| 123 | *Boophis* sp. CaNEW | Candidate | Yes |
| 124 | *Boophis andohahela* | Described | Yes |
| 125 | *Boophis* sp. 23 | Candidate | Yes |
| 126 | *Boophis* sp. aff. *tampoka* "North" | Candidate | Yes |
| 127 | *Boophis tampoka* | Described | Yes |
| 128 | *Boophis* sp. 24 | Candidate | Yes |
| 129 | *Boophis luteus* | Described | Yes |
| 130 | *Boophis* sp. CaNEW | Candidate | Yes |
| 131 | *Boophis* sp. aff. *luteus* "Andasibe" | Candidate | Yes |
| 132 | *Boophis englaenderi* | Described | Yes |
| 133 | *Boophis septentrionalis* | Described | Yes |
| 134 | *Boophis* sp. 36 | Candidate | Yes |
| 135 | *Boophis* sp. 37 | Candidate | Yes |
| 136 | *Boophis sandrae* | Described | Yes |
| 137 | *Boophis anjanaharibeensis* | Described | Yes |
| 138 | *Boophis jaegeri* | Described | Yes |
| 139 | *Boophis andreonei* | Described | Yes |
| 140 | *Boophis elenae* | Described | Yes |
| 141 | *Boophis* sp. 21 | Candidate | Yes |
| 142 | *Boophis* sp. aff. *elenae* | Candidate | No |
| 143 | *Boophis rappiodes* | Described | Yes |
| 144 | *Boophis* sp. aff. *rappiodes* "Ranomafana" | Candidate | Yes |
| 145 | *Boophis* sp. aff. *rappiodes* "Ranomafana" | Candidate | Yes |
| 146 | *Boophis ankarafensis* | Described | Yes |
| 147 | *Boophis bottae* | Described | Yes |
| 148 | *Boophis* sp. aff. *bottae* "Ranomafana" | Candidate | Yes |
| 149 | *Boophis* sp. CaNEW | Candidate | Yes |
| 150 | *Boophis erythrodactylus* | Described | Yes |
| 151 | *Boophis tasymena* | Described | Yes |
| 152 | *Boophis viridis* | Described | Yes |
| 153 | *Boophis williamsi* | Described | Yes |
| 154 | *Boophis* sp. Ca33 | Candidate | Yes |
| 155 | *Boophis microtympanum* | Described | Yes |
| 156 | *Boophis laurenti* | Described | Yes |
| 157 | *Boophis rhodoscelis* | Described | Yes |
| 158 | *Boophis andrangoloaka* | Described | Yes |
| 159 | *Boophis majori* | Described | Yes |
| 160 | *Boophis narinsi* | Described | Yes |
| 161 | *Boophis lilianae* | Described | Yes |
| 162 | *Boophis baetkei* | Described | Yes |
| 163 | *Boophis ulftunni* | Described | Yes |
| 164 | *Boophis* sp. aff. *ulftunni "*Marojejy" | Candidate | Yes |
| 165 | *Boophis* sp. CaNEW | Candidate | Yes |
| 166 | *Boophis pyrrhus* | Described | Yes |
| 167 | *Boophis* sp. aff. *pyrrhus* "Sahavontsira" | Candidate | Yes |
| 168 | *Boophis haematopus* | Described | Yes |
| 169 | *Boophis picturatus* | Described | Yes |
| 170 | *Boophis* sp. aff. *picturatus* "Ranomafana" | Candidate | Yes |
| 171 | *Boophis* sp. aff. *picturatus* "Ste Luce" | Candidate | Yes |
| 172 | *Boophis feonnyala* | Described | Yes |
| 173 | *Boophis miniatus* | Described | Yes |
| 174 | *Boophis piperatus* | Described | Yes |
| 175 | *Boophis arcanus* | Described | Yes |
| 176 | *Boophis* sp. 44 | Candidate | Yes |
| 177 | *Spinomantis bertini* | Described | Yes |
| 178 | *Spinomantis beckei* | Described | Yes |
| 179 | *Spinomantis* sp. 7 | Candidate | Yes |
| 180 | *Spinomantis guibei* | Described | Yes |
| 181 | *Spinomantis elegans* | Described | Yes |
| 182 | *Spinomantis microtis* | Described | Yes |
| 183 | *Spinomantis peraccae* | Described | Yes |
| 184 | *Spinomantis* sp. 4 | Candidate | Yes |
| 185 | *Spinomantis* sp. 10 | Candidate | Yes |
| 186 | *Spinomantis* sp. 5 | Candidate | Yes |
| 187 | *Spinomantis* sp. 11 | Candidate | Yes |
| 188 | *Spinomantis massi* | Described | Yes |
| 189 | *Spinomantis phantasticus* | Described | Yes |
| 190 | *Spinomantis* sp. 2 | Candidate | Yes |
| 191 | *Spinomantis tavaratra* | Described | Yes |
| 192 | *Spinomantis fimbriatus* | Described | Yes |
| 193 | *Spinomantis* sp. 9 | Candidate | Yes |
| 194 | *Spinomantis* sp. aff. *aglavei* | Candidate | Yes |
| 195 | *Spinomantis aglavei* | Described | Yes |
| 196 | *Spinomantis* sp. aff. *aglavei* "North" | Candidate | Yes |
| 197 | *Spinomantis* sp. 8 | Candidate | Yes |
| 198 | *Spinomantis* sp. 3 | Candidate | Yes |
| 199 | *Spinomantis* sp. CaNEW aff. *peraccae* | Candidate | Yes |
| 200 | *Spinomantis* sp. CaNEW | Candidate | Yes |
| 201 | *Spinomantis* sp. aff. *aglavei* | Candidate | Yes |
| 202 | *Spinomantis* sp. aff. *massi* | Candidate | Yes |
| 203 | *Spinomantis mirus* | Described | Yes |
| 204 | *Spinomantis brunae* | Described | No |
| 205 | *Spinomantis nussbaumi* | Described | No |
| 206 | *Mantella crocea* | Described | Yes |
| 207 | *Mantella milotympanum* | Described | Yes |
| 208 | *Mantella aurantiaca* | Described | Yes |
| 209 | *Mantella madagascariensis* | Described | Yes |
| 210 | *Mantella* sp. aff. *madagascariensis* "North" | Candidate | Yes |
| 211 | *Mantella pulchra* | Described | Yes |
| 212 | *Mantella bernhardi* | Described | Yes |
| 213 | *Mantella* sp. aff. *bernhardi* | Candidate | Yes |
| 214 | *Mantella haraldmeieri* | Described | Yes |
| 215 | *Mantella cowani* | Described | Yes |
| 216 | *Mantella baroni* | Described | Yes |
| 217 | *Mantella nigricans* | Described | Yes |
| 218 | *Mantella laevigata* | Described | Yes |
| 219 | *Mantella manery* | Described | Yes |
| 220 | *Mantella ebenaui* | Described | Yes |
| 221 | *Mantella viridis* | Described | Yes |
| 222 | *Mantella expectata* | Described | Yes |
| 223 | *Mantella* sp. aff. *betsileo* 2 | Candidate | Yes |
| 224 | *Mantella betsileo* | Described | Yes |
| 225 | *Mantella* sp. aff. *betsileo* 1 | Candidate | Yes |
| 227 | *Guibemantis depressiceps* | Described | Yes |
| 228 | *Guibemantis* sp. 18 | Candidate | Yes |
| 229 | *Guibemantis* sp. 19 | Candidate | Yes |
| 230 | *Guibemantis tornieri* | Described | Yes |
| 231 | *Guibemantis* sp. aff. *tornieri* | Candidate | Yes |
| 232 | *Guibemantis kathrinae* | Described | Yes |
| 233 | *Guibemantis* sp. aff. *timidus* | Candidate | Yes |
| 234 | *Guibemantis diphonus* | Described | Yes |
| 235 | *Guibemantis timidus* | Described | Yes |
| 236 | *Guibemantis methueni* | Described | Yes |
| 237 | *Guibemantis* sp. NEW | Candidate | Yes |
| 238 | *Guibemantis bicalcaratus* | Described | Yes |
| 239 | *Guibemantis* sp. 10 | Candidate | Yes |
| 240 | *Guibemantis* sp. 11 | Candidate | Yes |
| 241 | *Guibemantis* sp. 3 | Candidate | Yes |
| 242 | *Guibemantis* sp. aff. *bicalcaratus* "Betampona" | Candidate | Yes |
| 243 | *Guibemantis* sp. aff. *bicalcaratus* "Masoala" | Candidate | Yes |
| 244 | *Guibemantis flavobrunneus* | Described | Yes |
| 245 | *Guibemantis* sp. aff. *flavobrunneus* | Candidate | Yes |
| 246 | *Guibemantis pulcher* | Described | Yes |
| 247 | *Guibemantis* sp. aff. *pulcher* "Makira" | Candidate | Yes |
| 248 | *Guibemantis annulatus* | Described | Yes |
| 249 | *Guibemantis* sp. aff. *liber* "Mandraka" | Described | Yes |
| 250 | *Guibemantis* sp. aff. *liber* | Candidate | Yes |
| 251 | *Guibemantis* sp. aff. *liber* | Candidate | Yes |
| 252 | *Guibemantis* sp. aff. *liber* "Ranomafana" | Candidate | Yes |
| 253 | *Guibemantis* sp. aff. *liber* | Candidate | Yes |
| 254 | *Guibemantis* sp. aff. *liber* 1 | Described | Yes |
| 255 | *Guibemantis* sp. aff. *liber* "Makira1" | Candidate | No |
| 256 | *Guibemantis* sp. aff. *liber* "Makira2" | Candidate | Yes |
| 257 | *Guibemantis* sp. aff. *liber* | Candidate | Yes |
| 258 | *Guibemantis* sp. aff. *liber* | Candidate | Yes |
| 259 | *Guibemantis* sp. aff. *liber* "MarojejyAndapa" | Candidate | Yes |
| 260 | *Guibemantis punctatus* | Described | Yes |
| 261 | *Guibemantis* sp. 14 | Candidate | Yes |
| 262 | *Guibemantis albomaculatus* | Candidate | Yes |
| 263 | *Guibemantis* sp. 13 | Candidate | Yes |
| 264 | *Guibemantis milingilingy* | Described | Yes |
| 265 | *Guibemantis* sp. aff. *liber* "Maroantsetra" | Candidate | Yes |
| 266 | *Guibemantis* sp. aff. *punctatus* new "Betampona" | Candidate | Yes |
| 267 | *Guibemantis albolineatus* | Described | Yes |
| 268 | *Guibemantis* sp. aff. *bicalcaratus* CaNEW "Marojejy3" | Candidate | Yes |
| 269 | *Guibemantis* sp. 16 | Candidate | Yes |
| 270 | *Guibemantis woosteri* | Described | Yes |
| 271 | *Guibemantis* sp. 8 | Candidate | Yes |
| 272 | *Guibemantis wattersoni* | Described | Yes |
| 273 | *Guibemantis* sp. 1 | Candidate | Yes |
| 274 | *Guibemantis* sp. 2 | Candidate | Yes |
| 275 | *Guibemantis* sp. aff. *tasifotsy* | Candidate | Yes |
| 276 | *Guibemantis tasifotsy* | Described | Yes |
| 277 | *Wakea madinika* | Described | Yes |
| 278 | *Tsingymantis antitra* | Described | Yes |
| 279 | *Laliostoma labrosum* | Described | Yes |
| 280 | *Gephyromantis* sp. aff. *silvanus* | Candidate | Yes |
| 281 | *Gephyromantis* sp. 15 | Candidate | Yes |
| 282 | *Gephyromantis silvanus* | Described | Yes |
| 283 | *Gephyromantis* sp. 23 | Candidate | Yes |
| 284 | *Gephyromantis webbi* | Described | Yes |
| 285 | *Gephyromantis* sp. aff. *webbi* | Candidate | Yes |
| 286 | *Gephyromantis rivicola* | Described | Yes |
| 287 | *Gephyromantis lomorina* | Described | Yes |
| 288 | *Gephyromantis* sp. aff. *webbi* "Antanambe" | Candidate | Yes |
| 289 | *Gephyromantis horridus* | Described | Yes |
| 290 | *Gephyromantis ranjomavo* | Described | Yes |
| 291 | *Gephyromantis* sp. aff. *horridus* | Candidate | Yes |
| 292 | *Gephyromantis* sp. 12 | Candidate | Yes |
| 293 | *Gephyromantis ventrimaculatus* | Described | Yes |
| 294 | *Gephyromantis* sp. aff. *malagasius* | Candidate | Yes |
| 295 | *Gephyromantis* sp. 14 | Candidate | Yes |
| 296 | *Gephyromantis malagasius* | Described | Yes |
| 297 | *Gephyromantis* sp. aff. *malagasius* | Candidate | Yes |
| 298 | *Gephyromantis* sp. 13 | Candidate | Yes |
| 299 | *Gephyromantis striatus* | Described | Yes |
| 301 | *Gephyromantis* sp. aff. *luteus* "Marojejy" | Candidate | Yes |
| 302 | *Gephyromantis plicifer* | Described | Yes |
| 303 | *Gephyromantis pedronoi* | Described | Yes |
| 304 | *Gephyromantis* sp. aff. *luteus* "Masoala" | Candidate | Yes |
| 305 | *Gephyromantis sculpturatus* "Vevembe" | Described | Yes |
| 306 | *Gephyromantis* sp. aff. *sculpturatus* | Candidate | Yes |
| 307 | *Gephyromantis* sp. aff. *luteus* | Candidate | Yes |
| 308 | *Gephyromantis luteus* | Described | Yes |
| 309 | *Gephyromantis* sp. aff*. luteus* | Candidate | Yes |
| 310 | *Gephyromantis moseri* | Described | Yes |
| 311 | *Gephyromantis* sp. aff. *moseri* "Makira" | Candidate | Yes |
| 312 | *Gephyromantis* sp. aff. *moseri* "Masoala" | Candidate | Yes |
| 313 | *Gephyromantis* sp. 22 | Candidate | Yes |
| 314 | *Gephyromantis* sp. 19 | Candidate | Yes |
| 315 | *Gephyromantis* sp. 18 | Candidate | Yes |
| 316 | *Gephyromantis cornutus* | Described | Yes |
| 317 | *Gephyromantis* sp. aff. *cornutus* "Ivohibe" | Candidate | Yes |
| 318 | *Gephyromantis tschenki* | Described | Yes |
| 319 | *Gephyromantis* sp. aff. *cornutus* | Candidate | Yes |
| 320 | *Gephyromantis redimitus* "Ambohitsara" | Described | Yes |
| 321 | *Gephyromantis redimitus* "Betampona" | Described | Yes |
| 322 | *Gephyromantis redimitus* "Vohidrazana" | Described | Yes |
| 323 | *Gephyromantis redimitus* "Andranobe" | Described | Yes |
| 324 | *Gephyromantis redimitus* "Nosy Mangabe" | Described | Yes |
| 325 | *Gephyromantis leucomaculatus* | Described | Yes |
| 326 | *Gephyromantis granulatus* | Described | Yes |
| 327 | *Gephyromantis schilfi* | Described | Yes |
| 328 | *Gephyromantis* sp. 16 | Candidate | Yes |
| 329 | *Gephyromantis* sp. 17 | Candidate | Yes |
| 330 | *Gephyromantis* sp. aff. *leucomaculatus* | Candidate | Yes |
| 331 | *Gephyromantis zavona* | Described | Yes |
| 333 | *Gephyromantis saturnini* | Described | Yes |
| 334 | *Gephyromantis* sp. aff. *saturnini* | Candidate | Yes |
| 335 | *Gephyromantis salegy* | Described | Yes |
| 336 | *Gephyromantis tandroka* | Described | Yes |
| 337 | *Gephyromantis grosjeani* | Described | Yes |
| 338 | *Gephyromantis tohatra* | Described | Yes |
| 339 | *Gephyromantis asper* | Described | Yes |
| 340 | *Gephyromantis tahotra* | Described | Yes |
| 341 | *Gephyromantis ceratophrys* | Described | Yes |
| 342 | *Gephyromantis spinifer* | Described | Yes |
| 343 | *Gephyromantis angano* | Described | Yes |
| 344 | *Gephyromantis* sp. 29 | Candidate | Yes |
| 345 | *Gephyromantis ambohitra* | Candidate | Yes |
| 346 | *Gephyromantis* sp. aff. *ambohitra* | Candidate | Yes |
| 347 | *Gephyromantis kintana* | Described | Yes |
| 348 | *Gephyromantis atsingy* | Described | Yes |
| 349 | *Gephyromantis corvus* | Described | Yes |
| 350 | *Gephyromantis* sp. aff. *corvus "*Makira" | Candidate | Yes |
| 351 | *Gephyromantis pseudoasper* | Described | Yes |
| 352 | *Gephyromantis leucocephalus* 1 | Described | Yes |
| 353 | *Gephyromantis leucocephalus* 2 | Described | Yes |
| 354 | *Gephyromantis* sp. 6 | Candidate | Yes |
| 355 | *Gephyromantis decaryi* | Described | Yes |
| 356 | *Gephyromantis hintelmannae* | Described | Yes |
| 357 | *Gephyromantis* sp. 7 | Candidate | Yes |
| 358 | *Gephyromantis verrucosus* | Described | Yes |
| 359 | *Gephyromantis thelenae* | Described | Yes |
| 360 | *Gephyromantis eiselti* | Described | Yes |
| 361 | *Gephyromantis mafy* | Described | Yes |
| 362 | *Gephyromantis enki* | Described | Yes |
| 363 | *Gephyromantis blanci* | Described | Yes |
| 364 | *Gephyromantis runewsweeki* | Described | Yes |
| 365 | *Gephyromantis* sp. aff. *boulengeri* "Ambohitsara" | Candidate | Yes |
| 366 | *Gephyromantis* sp. aff*. boulengeri* "Ranomafana" | Candidate | Yes |
| 367 | *Gephyromantis* sp. 4 | Candidate | Yes |
| 368 | *Gephyromantis* sp. 5 | Candidate | Yes |
| 369 | *Gephyromantis* sp. 24 | Candidate | Yes |
| 370 | *Gephyromantis* sp. aff. *boulengeri* "Nosy Mangabe" | Candidate | Yes |
| 371 | *Gephyromantis* sp. aff. *boulengeri* "Masoala" | Candidate | Yes |
| 372 | *Gephyromantis boulengeri* | Described | Yes |
| 373 | *Gephyromantis* sp. aff. *boulengeri* "Marolambo" | Candidate | Yes |
| 374 | *Gephyromantis* sp. aff. *boulengeri* "Manompana" | Candidate | Yes |
| 375 | *Gephyromantis* sp. "Masoala" | Candidate | No |
| 376 | *Gephyromantis klemmeri* | Described | Yes |
| 377 | *Mantidactylus* sp. 67 | Candidate | Yes |
| 378 | *Mantidactylus radaka* | Described | Yes |
| 379 | *Mantidactylus guttulatus* | Described | Yes |
| 380 | *Mantidactylus* sp. 55 | Candidate | Yes |
| 381 | *Mantidactylus* sp. 56 | Candidate | Yes |
| 382 | *Mantidactylus grandidieri* | Described | Yes |
| 384 | *Mantidactylus femoralis* | Described | Yes |
| 385 | *Mantidactylus* sp. aff. *femoralis* | Candidate | Yes |
| 386 | *Mantidactylus* sp. 38 | Candidate | Yes |
| 387 | *Mantidactylus* sp. 60 | Candidate | Yes |
| 388 | *Mantidactylus* sp. aff. *femoralis* | Candidate | Yes |
| 389 | *Mantidactylus* sp. 37 | Candidate | Yes |
| 390 | *Mantidactylus zolitschka* | Described | Yes |
| 391 | *Mantidactylus* sp. 42 | Candidate | Yes |
| 392 | *Mantidactylus* sp. 41 | Candidate | Yes |
| 393 | *Mantidactylus* sp. 40 | Candidate | Yes |
| 394 | *Mantidactylus* sp. 39 | Candidate | Yes |
| 395 | *Mantidactylus ambreensis* | Described | Yes |
| 396 | *Mantidactylus* sp. aff*. ambreensis* | Candidate | Yes |
| 397 | *Mantidactylus* sp. aff. *ambreensis* | Candidate | Yes |
| 398 | *Mantidactylus* sp. 44 | Candidate | Yes |
| 399 | *Mantidactylus* sp. 43 | Candidate | Yes |
| 400 | *Mantidactylus* sp. Ca64 | Candidate | Yes |
| 401 | *Mantidactylus* sp. 47 | Candidate | Yes |
| 402 | *Mantidactylus* sp. 45 | Candidate | Yes |
| 403 | *Mantidactylus* sp. 46 | Candidate | Yes |
| 404 | *Mantidactylus majori* "Saint Luce" | Candidate | Yes |
| 405 | *Mantidactylus majori* | Described | Yes |
| 406 | *Mantidactylus* sp. aff. *majori* | Candidate | Yes |
| 407 | *Mantidactylus* sp. CaNEW | Candidate | Yes |
| 408 | *Mantidactylus argenteus* | Described | Yes |
| 409 | *Mantidactylus cowanii* | Described | Yes |
| 410 | *Mantidactylus* sp. 48 | Candidate | Yes |
| 411 | *Mantidactylus* sp. 50 | Candidate | Yes |
| 412 | *Mantidactylus* sp. aff. *femolugubris* | Candidate | Yes |
| 413 | *Mantidactylus lugubris* 1 | Described | Yes |
| 414 | *Mantidactylus lugubris* 2 | Described | Yes |
| 415 | *Mantidactylus* sp. 49 | Candidate | Yes |
| 416 | *Mantidactylus petakorona* | Described | Yes |
| 417 | *Mantidactylus* sp. 53 | Candidate | Yes |
| 418 | *Mantidactylus* sp. 54 | Candidate | Yes |
| 420 | *Mantidactylus melanopleura* | Described | Yes |
| 421 | *Mantidactylus* sp. aff. *melanopleura* | Candidate | Yes |
| 422 | *Mantidactylus* sp. 13 | Candidate | Yes |
| 423 | *Mantidactylus* sp. aff. *melanopleura* "Manombo" | Candidate | Yes |
| 424 | *Mantidactylus* sp. CaNEW "Sahavontsira" | Candidate | Yes |
| 425 | *Mantidactylus zipperi* | Described | Yes |
| 426 | *Mantidactylus* sp. aff. *zipperi* | Candidate | Yes |
| 427 | *Mantidactylus* sp. aff. *opiparis* | Candidate | Yes |
| 428 | *Mantidactylus* sp. 3 | Candidate | Yes |
| 429 | *Mantidactylus opiparis* | Described | Yes |
| 430 | *Mantidactylus* sp. 5 | Candidate | Yes |
| 431 | *Mantidactylus* sp. 4 | Candidate | Yes |
| 432 | *Mantidactylus paidroa* | Described | Yes |
| 433 | *Mantidactylus delormei* | Described | Yes |
| 434 | *Mantidactylus brevipalmatus* | Described | Yes |
| 435 | *Mantidactylus aerumnalis* | Described | Yes |
| 436 | *Mantidactylus* sp. 1 | Candidate | Yes |
| 437 | *Mantidactylus albofrenatus* | Described | Yes |
| 438 | *Mantidactylus charlotteae* | Described | Yes |
| 439 | *Mantidactylus* sp. aff. *charlottae* | Candidate | Yes |
| 440 | *Mantidactylus* sp. 11 | Candidate | Yes |
| 441 | *Mantidactylus* sp. 12 | Candidate | Yes |
| 442 | *Mantidactylus* sp. aff. *zipperi* | Candidate | Yes |
| 443 | *Mantidactylus* sp. 9 | Candidate | Yes |
| 444 | *Mantidactylus* sp. aff*. albofrenatus* | Candidate | Yes |
| 445 | *Mantidactylus* sp. 10 | Candidate | Yes |
| 446 | *Mantidactylus* sp. 58 | Candidate | Yes |
| 447 | *Mantidactylus tricinctus* | Described | Yes |
| 448 | *Mantidactylus* sp. 73 | Candidate | Yes |
| 449 | *Mantidactylus* sp. 7 | Candidate | Yes |
| 450 | *Mantidactylus* sp. aff. *tricinctus* | Candidate | Yes |
| 451 | *Mantidactylus* sp. 6 | Candidate | Yes |
| 452 | *Mantidactylus* sp. aff. *tricinctus* | Candidate | Yes |
| 453 | *Mantidactylus* sp. aff. *tricinctus* | Candidate | Yes |
| 454 | *Mantidactylus* sp. 71 | Candidate | Yes |
| 455 | *Mantidactylus biporus* | Described | Yes |
| 456 | *Mantidactylus* sp. 17 | Candidate | Yes |
| 457 | *Mantidactylus* sp. 77 | Candidate | Yes |
| 458 | *Mantidactylus* sp. Ca81 | Candidate | Yes |
| 459 | *Mantidactylus* sp. 16 | Candidate | Yes |
| 460 | *Mantidactylus* sp. 15 | Candidate | Yes |
| 461 | *Mantidactylus* sp. 78 | Candidate | Yes |
| 462 | *Mantidactylus pauliani* | Described | Yes |
| 463 | *Mantidactylus alutus* | Described | Yes |
| 464 | *Mantidactylus* sp. 14 | Candidate | Yes |
| 465 | *Mantidactylus* sp. 23 | Candidate | Yes |
| 466 | *Mantidactylus* sp. Ca76 | Candidate | Yes |
| 467 | *Mantidactylus* sp. 24 | Candidate | Yes |
| 468 | *Mantidactylus* sp. 22 | Candidate | Yes |
| 469 | *Mantidactylus* sp. aff. *biporus* | Candidate | Yes |
| 470 | *Mantidactylus* sp. Ca21 | Candidate | Yes |
| 471 | *Mantidactylus curtus* | Described | Yes |
| 472 | *Mantidactylus bourgati* | Described | Yes |
| 473 | *Mantidactylus* sp. 18 | Candidate | Yes |
| 474 | *Mantidactylus madecassus* | Described | Yes |
| 475 | *Mantidactylus* sp. 19 | Candidate | Yes |
| 476 | *Mantidactylus* sp. 20 | Candidate | Yes |
| 477 | *Mantidactylus ulcerosus* | Described | Yes |
| 478 | *Mantidactylus bellyi* | Described | Yes |
| 479 | *Mantidactylus* sp. 31 | Candidate | Yes |
| 480 | *Mantidactylus* sp. 83 | Candidate | Yes |
| 481 | *Mantidactylus betsileanus* 1 | Described | Yes |
| 482 | *Mantidactylus* sp. 25 | Candidate | Yes |
| 483 | *Mantidactylus* sp. 26 | Candidate | Yes |
| 485 | *Mantidactylus* sp. 27 | Candidate | Yes |
| 486 | *Mantidactylus* sp. 34 | Candidate | Yes |
| 487 | *Mantidactylus* sp. 36 | Candidate | Yes |
| 488 | *Mantidactylus* sp. 35 | Candidate | Yes |
| 489 | *Mantidactylus schulzi* | Described | Yes |
| 490 | *Mantidactylus* sp. 33 | Candidate | Yes |
| 491 | *Mantidactylus betsileanus* 2 | Described | Yes |
| 492 | *Mantidactylus* sp. aff. *betsileanus* | Candidate | Yes |
| 494 | *Mantidactylus* sp. 30 | Candidate | Yes |
| 495 | *Mantidactylus noralottae* | Described | Yes |
| 496 | *Mantidactylus* sp. 29 | Candidate | Yes |
| 497 | *Mantidactylus* sp. 65 | Candidate | Yes |
| 498 | *Mantidactylus* sp. Ca28 | Candidate | Yes |
| 499 | *Mantidactylus* sp. CaNEW | Candidate | Yes |
| 500 | *Mantidactylus* sp. aff. *tricinctus* "Ambatobe" | Candidate | Yes |
| 501 | *Aglyptodactylus* sp. aff. *inguinalis* "Makay" | Candidate | Yes |
| 502 | *Mantidactylus* sp. CaNEW "Makay" | Candidate | No |
| 503 | *Boophis* sp. aff. *marojezensis* | Candidate | Yes |
| 504 | *Mantidactylus* sp. 66 | Candidate | No |


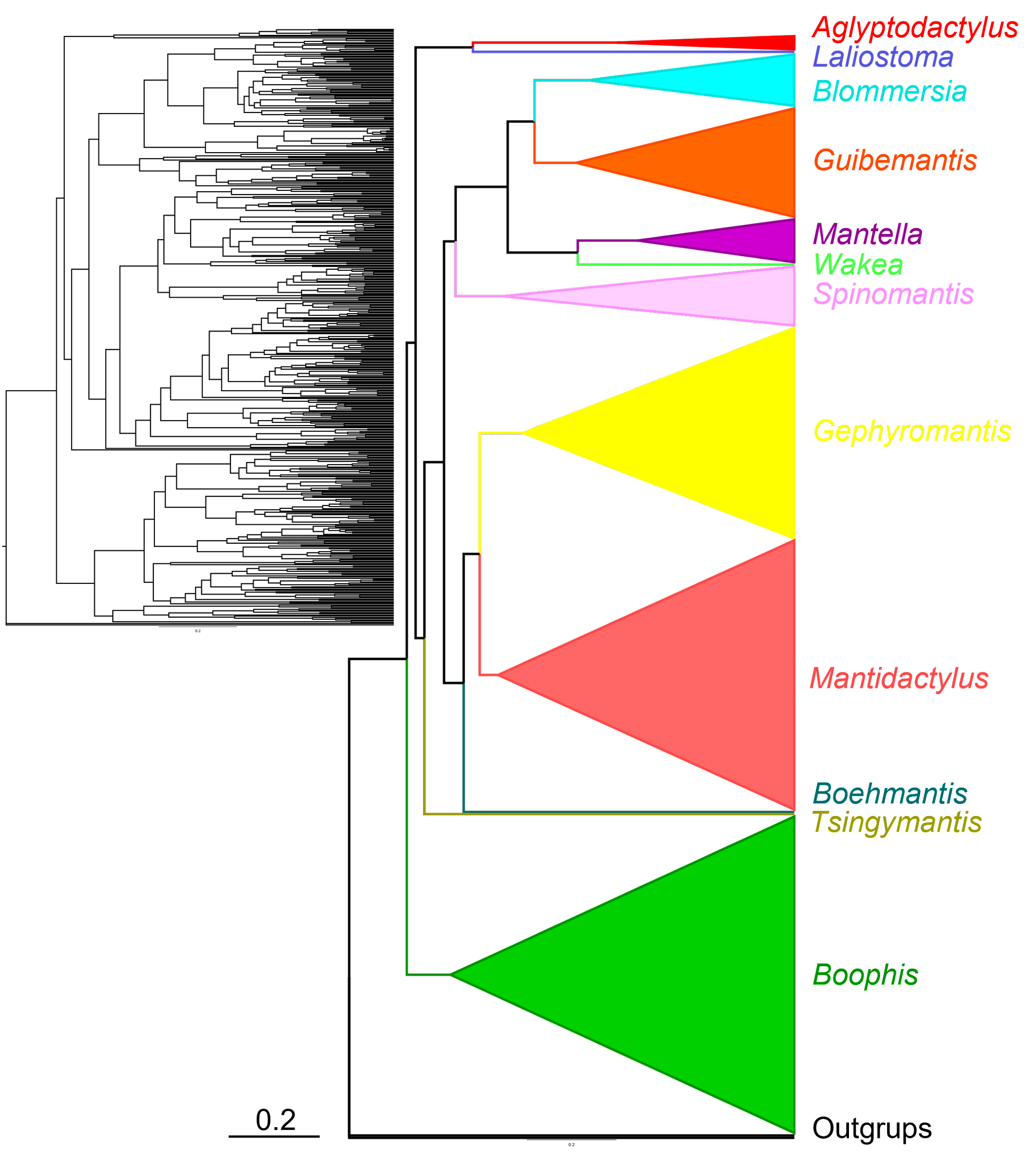


**Supplementary Figure S5 –** Topology used in this work ^6^ (after removing the two species of mantellid frogs endemic to the Comoros and the two outgroups), comprising 484 species and candidate species of mantellid frogs from Madagascar. Each coloured clade corresponds to a different genus of mantellid frogs, whose name is coloured accordingly. The raw topology of the tree is depicted in the top left corner.

**Supplementary Data S1 –** Original topology used in this work in newick format (note that *Blommersia transmarina* and *Boophis nauticus* from the Comoros and two outgroups are also include in this topology).

(OUT_Polypedates_spp:0.4026929,OUT_Heterixalus_variabilis:0.468443,(((((((5_Aglyptodactylus_madagascariensis:0.0488076,4_Aglyptodactylus_australis:0.02235236):0.01931967,((1_Aglyptodactylus_inguinalis:0.03050957,501_Aglyptodactylus_sp_aff_inguinalis_Makay:0.02940765):0.02371683,2_Aglyptodactylus_chorus:0.06051045):0.02506005):0.01042033,6_Aglyptodactylus_securifer:0.1002752):0.01416089,3_Aglyptodactylus_laticeps:0.06351476):0.07303023,279_Laliostoma_labrosum:0.1938446):0.03266474,(((((((((((9_Blommersia__sarotra:0.03656411,(7_Blommersia__sp_9:0.005504102,8_Blommersia__kely:0.019931):0.007781575):0.0142492,10_Blommersia__sp_8:0.03452124):0.01034205,15_Blommersia__sp_10:0.06139769):0.01957885,(11_Blommersia__angolafa:0.05379929,((12_Blommersia__grandisonae:0.03255058,30_Blommersia__sp_CaNEW:0.0288448):0.01202616,((14_Blommersia__sp_13:0.008485656,13_Blommersia__sp_6:0.01146382):0.01802895,31_Blommersia__sp_CaNEW:0.02509415):0.006170633):0.01359737):0.0166085):0.01166212,16_Blommersia__sp_11:0.06100784):0.02235549,(((19_Blommersia__wittei:0.05346854,28_Blommersia_transmarina:0.04865662):0.02699441,18_Blommersia__sp_5:0.04293094):0.01815627,(((20_Blommersia__blommersae:0.03539017,(21_Blommersia__sp_aff_blommersae_Ranomafana:0.0352065,29_Blommersia__sp_CaNEW:0.03488487):0.01421349):0.01425719,(((24_Blommersia__galani:0.03055677,23_Blommersia__variabilis:0.05671696):0.009509043,(26_Blommersia__dejongi:0.01714166,25_Blommersia__sp_aff_blommersae_Ivoloina:0.06142218):0.02427956):0.01194008,27_Blommersia__sp_12:0.04468032):0.01012413):0.01867683,22_Blommersia__domerguei:0.04699637):0.01555157):0.02544582):0.03065898,(((((236_Guibemantis_methueni:0.01022504,237_Guibemantis_sp_NEW:0.04074049):0.03622021,269_Guibemantis_sp_16:0.02706744):0.01605435,(270_Guibemantis_woosteri:0.02992061,268_Guibemantis_sp_aff_bicalcaratus_CaNEWMarojejy3:0.05255139):0.009009319):0.01322051,((((((251_Guibemantis_sp_aff_liber_:0.02661226,254_Guibemantis_sp_aff_liber_1:0.02706443):0.01111706,((249_Guibemantis_sp_aff_liber_Mandraka:0.01584761,258_Guibemantis_sp_aff_liber:0.02859881):0.005323679,((252_Guibemantis_sp_aff_liber_Ranomafana:0.0159334,253_Guibemantis_sp_aff_liber_:0.02377967):0.005112523,250_Guibemantis_sp_aff_liber:0.1047452):0.00561807):0.01311874):0.01193838,((273_Guibemantis_sp_1:0.0170249,274_Guibemantis_sp_2:0.04550543):0.03864449,(256_Guibemantis_sp_aff_liber_Makira2:0.01327291,259_Guibemantis_sp_aff_liber_Marojejy1_Andapa:0.02043971):0.02798771):0.03180347):0.007451663,((((246_Guibemantis_pulcher:0.02064655,247_Guibemantis_sp_aff_pulcher_Makira:0.02497793):0.01148891,(244_Guibemantis_flavobrunneus:0.01223223,245_Guibemantis_sp_aff_flavobrunneus:0.06104681):0.02529533):0.01050008,248_Guibemantis_annulatus:0.05158962):0.006763908,(267_Guibemantis_albolineatus:0.02895,261_Guibemantis_sp_14:0.0365621):0.01337235):0.002814977):0.003316843,(((((262_Guibemantis_albomaculatus:0.0034145,263_Guibemantis_sp_13:0.07853161):0.02096312,264_Guibemantis_milingilingy:0.03209681):0.01020179,((260_Guibemantis_punctatus:4.378049E-4,257_Guibemantis_sp_aff_liber:4.596033E-4):0.006704794,(265_Guibemantis_sp_aff_liber_Maroantsetra:0.01870718,266_Guibemantis_sp_aff_punctatus_new_Betampona:0.01835009):0.03045023):0.02242992):0.0270124,(275_Guibemantis_sp_aff_tasifotsy:0.02786948,276_Guibemantis_tasifotsy:0.01389875):0.04178495):0.004298666,(271_Guibemantis_sp_8:0.03305374,272_Guibemantis_wattersoni_:0.02810663):0.01826527):0.003286223):0.004642073,((241_Guibemantis_sp_3:0.02925099,(242_Guibemantis_sp_aff_bicalcaratus_Betampona:0.02476829,243_Guibemantis_sp_aff_bicalcaratus_Masoala:0.02166452):0.01163248):0.01302664,(238_Guibemantis_bicalcaratus:0.05992679,(239_Guibemantis_sp_10:0.00703576,240_Guibemantis_sp_11:0.03964601):0.02481933):0.01225317):0.008141663):0.00642472):0.03481191,(((((231_Guibemantis_sp_aff_tornieri:0.00313347,230_Guibemantis_tornieri:0.006505257):0.0191067,232_Guibemantis_kathrinae:0.01379836):0.0202944,(227_Guibemantis_depressiceps:0.01421848,228_Guibemantis_sp_18:0.02770012):0.02772367):0.007591717,229_Guibemantis_sp_19:0.05216075):0.02692419,((233_Guibemantis_sp_aff_timidus:0.04187864,235_Guibemantis_timidus:0.02138823):0.01524551,234_Guibemantis_diphonus:0.02508116):0.02881806):0.01465):0.03705144):0.0264155,(((((207_Mantella_milotympanum:9.578198E-4,206_Mantella_crocea:0.00202937):0.008551017,(((209_Mantella_madagascariensis:0.003878807,210_Mantella_sp_aff_madagascariensis_North:0.004833458):0.004038485,211_Mantella_pulchra:0.009769799):0.006071485,208_Mantella_aurantiaca:0.01183944):0.004041994):0.04390213,(((220_Mantella_ebenaui:0.001850358,221_Mantella_viridis:0.004561842):0.007991719,(((222_Mantella_expectata:0.008376707,225_Mantella_sp_aff_betsileo_1:0.007853296):0.002645921,223_Mantella_sp_aff_betsileo_2:0.007329488):0.01342026,224_Mantella_betsileo:0.007754429):0.005853322):0.02923956,(218_Mantella_laevigata:0.03134214,219_Mantella_manery:0.03198827):0.0330808):0.01948821):0.01681683,((212_Mantella_bernhardi:0.02066408,213_Mantella_sp_aff_bernhardi:0.03967261):0.04698409,((214_Mantella_haraldmeieri:0.006716825,(216_Mantella_baroni:0.001470586,217_Mantella_nigricans:0.002019338):0.001639918):0.00354921,215_Mantella_cowani:0.008373982):0.04506991):0.0108345):0.03224493,277_Wakea_madinika:0.1478838):0.0443482):0.05397279,(((((178_Spinomantis_beckei:0.04210331,177_Spinomantis_bertini:0.04212912):0.05404744,(179_Spinomantis_sp_7:0.06862437,203_Spinomantis_mirus:0.04374181):0.02562275):0.02679432,180_Spinomantis_guibei:0.07867632):0.01629952,182_Spinomantis_microtis:0.06589653):0.01032136,(181_Spinomantis_elegans:0.06092883,(((((((183_Spinomantis_peraccae:0.04637083,185_Spinomantis_sp_10:0.01479373):0.00846024,186_Spinomantis_sp_5:0.01540311):0.005763728,184_Spinomantis_sp_4:0.02503076):0.005909334,((198_Spinomantis_sp_3:0.0265709,199_Spinomantis_sp_CaNEW_aff_peraccae:0.05260119):0.01991397,200_Spinomantis__sp_CaNEW:0.04739643):0.0337319):0.006083008,187_Spinomantis_sp_11:0.02578877):0.02331023,(((((((195_Spinomantis_aglavei:0.01079803,196_Spinomantis_sp_aff_aglavei_North:0.02085606):0.007259337,(194_Spinomantis_sp_aff_aglavei_:0.01762198,201_Spinomantis_sp_aff_aglavei:0.0268061):0.01910178):0.03540482,(192_Spinomantis_fimbriatus:0.03348226,193_Spinomantis_sp_9:0.04117513):0.01766483):0.01321918,191_Spinomantis_tavaratra:0.02660897):0.01511523,190_Spinomantis_sp_2:0.05326119):0.01194679,202_Spinomantis_sp_aff_massi:0.06576218):0.01295212,(188_Spinomantis_massi:0.0451272,189_Spinomantis_phantasticus:0.04348248):0.05249685):0.007318052):0.01369731,197_Spinomantis_sp_8:0.1027649):0.011372):0.02391911):0.0262773):0.01262983,(((((((((282_Gephyromantis_silvanus:0.04397827,283_Gephyromantis_sp_23:0.04650252):0.01188608,(286_Gephyromantis_rivicola:0.04662375,(280_Gephyromantis_sp_aff_silvanus:0.06078319,281_Gephyromantis_sp_15:0.05918716):0.02635793):0.01007373):0.02763609,((284_Gephyromantis_webbi:0.02899138,285_Gephyromantis_sp_aff_webbi:0.02265258):0.02634248,288_Gephyromantis_sp_aff_webbi_Antanambe:0.01083966):0.05975795):0.02174233,((376_Gephyromantis_klemmeri:0.07620447,287_Gephyromantis_lomorina:0.08968327):0.02137329,((((((298_Gephyromantis_sp_13:0.04217778,(296_Gephyromantis_malagasius:0.01444823,297_Gephyromantis_sp_aff_malagasius:0.04763791):0.0252145):0.01262859,295_Gephyromantis_sp_14:0.03567509):0.01798803,294_Gephyromantis_sp_aff_malagasius:0.05667274):0.01517647,299_Gephyromantis_striatus:0.08760534):0.006993038,293_Gephyromantis_ventrimaculatus:0.06153613):0.007495727,(((289_Gephyromantis_horridus:0.02403235,291_Gephyromantis_sp_aff_horridus:0.01180763):0.01330988,290_Gephyromantis_ranjomavo:0.02769869):0.02616724,292_Gephyromantis_sp_12:0.0741249):0.03308352):0.01906917):0.01313597):0.01506445,(((((306_Gephyromantis_sp_aff_sculpturatus:0.01089276,305_Gephyromantis_sculpturatus_Vevembe:0.004916957):0.02276604,((308_Gephyromantis_luteus:0.01937299,309_Gephyromantis_sp_aff_luteus:0.0225624):0.01331888,307_Gephyromantis_sp_aff_luteus:0.03396582):0.01026628):0.00878218,(301_Gephyromantis_sp_aff_luteus_Marojejy:0.02833841,304_Gephyromantis_sp_aff_luteus_Masoala:0.03343553):0.01764374):0.07591247,(302_Gephyromantis_sp_21:0.07024662,303_Gephyromantis_plicifer:0.08174192):0.04402398):0.04056811,(((((316_Gephyromantis_cornutus:0.009202701,(318_Gephyromantis_tschenki:0.0102434,319_Gephyromantis_sp_aff_cornutus:0.02280595):0.01258136):0.005406633,317_Gephyromantis_sp_aff_cornutus_Ivohibe:0.02426106):0.04141394,((((322_Gephyromantis_redimitus_Vohidrazana:0.01279588,321_Gephyromantis_redimitus_Betampona:0.03682077):0.007825207,323_Gephyromantis_redimitus_Andranobe:0.04213007):0.01529283,320_Gephyromantis_redimitus_Ambohitsara:0.0205391):0.01274508,324_Gephyromantis_redimitus_Nosy_Mangabe:0.04125172):0.02675198):0.04598019,((((333_Gephyromantis_saturnini:0.02221532,327_Gephyromantis_schilfi:0.02086474):0.01346877,((338_Gephyromantis_tohatra:0.01707092,(337_Gephyromantis_grosjeani:0.04700993,336_Gephyromantis_tandroka:0.01802329):0.02119904):0.003190331,(334_Gephyromantis_sp_aff_saturnini:0.03786952,335_Gephyromantis_salegy:0.0294675):0.007549579):0.006644108):0.04136636,((325_Gephyromantis_leucomaculatus:0.03629386,329_Gephyromantis_sp_17:0.04003654):0.01817714,(328_Gephyromantis_sp_16:0.04100723,330_Gephyromantis_sp_aff_leucomaculatus:0.01739639):0.0144967):0.03343584):0.008703873,(((310_Gephyromantis_moseri:0.01698418,(311_Gephyromantis__sp_aff_moseri_Makira:0.01886213,313_Gephyromantis_sp_22:0.01090331):0.01015915):0.02317665,312_Gephyromantis__sp_aff_moseri_Masoala:0.03004156):0.01991427,(314_Gephyromantis_sp_19:0.05564647,315_Gephyromantis_sp_18:0.07866738):0.02707933):0.03495514):0.006310408):0.005524828,(326_Gephyromantis_granulatus:0.07743339,331_Gephyromantis_zavona:0.08090727):0.0157626):0.02392762):0.01308766):0.009505817,(((((339_Gephyromantis_asper:0.05320803,342_Gephyromantis_spinifer:0.05706646):0.01067387,341_Gephyromantis_ceratophrys:0.04869071):0.01009751,340_Gephyromantis_tahotra:0.09084349):0.006655111,(343_Gephyromantis_angano:0.02150789,344_Gephyromantis_sp_29:0.002770383):0.02399051):0.02250558,(345_Gephyromantis_ambohitra:0.0505894,346_Gephyromantis_sp_aff_ambohitra:0.02978689):0.05056718):0.02573579):0.005269867,(((((((369_Gephyromantis_sp_24:0.03197969,((365_Gephyromantis_sp_aff_boulengeri_Ambohitsara:0.03488231,366_Gephyromantis_sp_aff_boulengeri_Ranomafana:0.03322274):0.01716517,373_Gephyromantis_sp_aff_boulengeri_Marolambo:0.02714766):0.01626178):0.01130043,(((370_Gephyromantis_sp_aff_boulengeri_Nosy_Mangabe:0.02312308,371_Gephyromantis_sp_aff_boulengeri_Masoala:0.0137254):0.01174197,374_Gephyromantis_sp_aff_boulengeri_Manompana:0.02006202):0.01206537,372_Gephyromantis_boulengeri:0.02052223):0.01664319):0.0231473,(((362_Gephyromantis_enki:0.02306133,364_Gephyromantis_runewsweeki:0.01989969):0.01248738,363_Gephyromantis_blanci:0.02682625):0.02439421,367_Gephyromantis_sp_4:0.02390201):0.008180596):0.006625223,368_Gephyromantis_sp_5:0.05202621):0.004779113,((359_Gephyromantis_thelenae:0.01910597,360_Gephyromantis_eiselti:0.01423662):0.01010117,361_Gephyromantis_mafy:0.02544142):0.03499592):0.006215651,((((352_Gephyromantis_leucocephalus_1:0.01451355,353_Gephyromantis_leucocephalus_2:0.02392515):0.006181312,354_Gephyromantis_sp_6:0.01583808):0.02271861,(355_Gephyromantis_decaryi:0.009906792,(358_Gephyromantis_verrucosus:0.02045917,356_Gephyromantis_hintelmannae:0.02117622):0.01456714):0.01399829):0.009259055,357_Gephyromantis_sp_7:0.0363371):0.01147532):0.07136329,(351_Gephyromantis_pseudoasper:0.109539,((347_Gephyromantis_kintana:0.05750607,(349_Gephyromantis_corvus:0.02309262,350_Gephyromantis_sp_aff_corvus_Makira:0.0308941):0.04672288):0.03310658,348_Gephyromantis_atsingy:0.08533418):0.02286668):0.03589128):0.008399422):0.03626179,(((((((((((((429_Mantidactylus_opiparis:0.02228196,((430_Mantidactylus_sp_5:0.02648764,431_Mantidactylus_sp_4:0.04757515):0.01274986,((433_Mantidactylus_delormei:0.01978957,434_Mantidactylus_brevipalmatus:0.01621081):0.03409602,432_Mantidactylus_paidroa:0.05120319):0.01337062):0.007924486):0.009426216,((420_Mantidactylus_melanopleura:0.02438426,(421_Mantidactylus_sp_aff_melanopleura:0.009508106,423_Mantidactylus_sp_aff_melanopleura_Manombo:0.02297724):0.01327937):0.02552739,(((424_Mantidactylus_sp_CaNEW_Sahavontsira:0.06394376,427_Mantidactylus_sp_aff_opiparis:0.01651333):0.01453132,425_Mantidactylus_zipperi:0.02723009):0.009277056,428_Mantidactylus_sp_3:0.01264975):0.02466396):0.0186629):0.006715408,422_Mantidactylus_sp_13:0.05121443):0.006593402,((440_Mantidactylus_sp_11:0.04208501,(442_Mantidactylus_sp_aff_zipperi:0.05372398,441_Mantidactylus_sp_12:0.0792929):0.009964139):0.004785118,(((444_Mantidactylus_sp_aff_albofrenatus:0.02715287,(438_Mantidactylus_charlotteae:0.02713377,439_Mantidactylus_sp_aff_charlottae:0.01070503):0.01401919):0.005450075,445_Mantidactylus_sp_10:0.02400321):0.008313442,(437_Mantidactylus_albofrenatus:0.04515722,443_Mantidactylus_sp_9:0.04478453):0.007343856):0.01418287):0.009419516):0.004978279,(435_Mantidactylus_aerumnalis:0.06766913,436_Mantidactylus_sp_1:0.06260243):0.03742983):0.008387009,426_Mantidactylus_sp_aff_zipperi_:0.02782371):0.01521219,454_Mantidactylus_sp_71:0.03997361):0.01415448,((((((((((((483_Mantidactylus_sp_26:0.0114247,482_Mantidactylus_sp_25:0.02658004):0.006201468,485_Mantidactylus_sp_27:0.01427616):0.03724645,481_Mantidactylus_betsileanus:0.04438283):0.008438771,486_Mantidactylus_sp_34:0.04908538):0.008308317,(492_Mantidactylus_sp_aff_betsileanus:0.0166293,491_Mantidactylus_betsileanus:0.02242459):0.02464919):0.008039249,(494_Mantidactylus_sp_30:0.02654002,(((495_Mantidactylus_noralottae:0.01559335,497_Mantidactylus_sp_65:0.02619272):0.008176,(498_Mantidactylus_sp_Ca28:0.005859758,499_Mantidactylus_sp_CaNEW:0.04936775):0.01868817):0.005034773,496_Mantidactylus_sp_29:0.01740966):0.009219241):0.0234172):0.005069475,(487_Mantidactylus_sp_36:0.01901997,488_Mantidactylus_sp_35:0.02602927):0.05190156):0.01073447,479_Mantidactylus_sp_31:0.0712193):0.00964075,480_Mantidactylus_sp_83:0.04471533):0.008509662,((477_Mantidactylus_ulcerosus:0.03956831,478_Mantidactylus_bellyi:0.03061479):0.03872623,(489_Mantidactylus_schulzi:0.02585448,490_Mantidactylus_sp_33:0.03078586):0.04334838):0.01208734):0.01504189,(((((((((470_Mantidactylus__sp_Ca21:0.02336729,468_Mantidactylus_sp_22:0.02826209):0.02517209,(466_Mantidactylus_sp_Ca76:0.03273082,469_Mantidactylus_sp_aff_biporus:0.0245252):0.0253054):0.01671031,467_Mantidactylus_sp_24:0.04168829):0.008169938,465_Mantidactylus_sp_23:0.05906968):0.01104728,462_Mantidactylus_pauliani:0.02654224):0.005770234,464_Mantidactylus_sp_14:0.1151156):0.005212737,(((473_Mantidactylus_sp_18:0.01708143,(474_Mantidactylus_madecassus:0.02370723,(475_Mantidactylus_sp_19:0.002000623,476_Mantidactylus_sp_20:0.006502267):0.009857893):0.004597404):0.01627181,472_Mantidactylus_bourgati:0.06511337):0.007064964,471_Mantidactylus_curtus:0.05652719):0.01393213):0.004649984,(((455_Mantidactylus_biporus:0.03775448,(456_Mantidactylus_sp_17:0.02559362,458_Mantidactylus_sp_Ca81:0.02485699):0.004591681):0.01608354,457_Mantidactylus_sp_77:0.01137445):0.01201636,((459_Mantidactylus_sp_16:0.01311251,460_Mantidactylus_sp_15:0.02541324):0.0143614,461_Mantidactylus_sp_78:0.02576364):0.007676212):0.0115824):0.003637686,463_Mantidactylus_alutus:0.04851146):0.02594661):0.005798727,(447_Mantidactylus_tricinctus:0.04863317,(((449_Mantidactylus_sp_7:0.01540063,450_Mantidactylus_sp_aff_tricinctus:0.03024748):0.0201641,((448_Mantidactylus_sp_73:0.01031441,453_Mantidactylus_sp_aff_tricinctus_:0.008698139):0.01762571,(500_Mantidactylus_sp_aff_tricinctus_Ambatobe:0.004820591,451_Mantidactylus_sp_6:0.01302457):0.01570186):0.01379471):0.01575018,452_Mantidactylus_sp_aff_tricinctus:0.04638826):0.01059345):0.0313941):0.01033234):0.007031687,446_Mantidactylus_sp_58:0.05716371):0.03036869,(404_Mantidactylus_majori_Saint_Luce:0.03098031,((405_Mantidactylus_majori:0.02699423,407_Mantidactylus_sp_CaNEW:0.09155312):0.008108554,406_Mantidactylus_sp_aff_majori:0.02789573):0.01775598):0.06094256):0.01029066,(((((386_Mantidactylus_sp_38:0.01138399,(387_Mantidactylus_sp_60:0.01099018,385_Mantidactylus_sp_aff_femoralis:0.04449453):0.004545477):0.01020129,(384_Mantidactylus_femoralis:0.0147697,(389_Mantidactylus_sp_37:0.01455376,388_Mantidactylus__sp_aff_femoralis:0.02596606):0.008347877):0.007783671):0.003685207,394_Mantidactylus_sp_39:0.01533017):0.02660217,((390_Mantidactylus_zolitschka:0.02709418,(391_Mantidactylus_sp_42:0.02577423,(393_Mantidactylus_sp_40:0.03171682,392_Mantidactylus_sp_41:0.03120954):0.008015241):0.004566141):0.01310037,((((398_Mantidactylus_sp_44:0.02605414,399_Mantidactylus_sp_43:0.03256335):0.0226118,(402_Mantidactylus_sp_45:0.03712716,403_Mantidactylus_sp_46:0.03597644):0.006880681):0.00570853,(400_Mantidactylus__sp_Ca64:0.03159937,401_Mantidactylus_sp_47:0.02970329):0.01408085):0.005650905,((395_Mantidactylus_ambreensis:0.007785899,397_Mantidactylus_sp_aff_ambreensis:0.05229745):0.02021248,396_Mantidactylus__sp_aff_ambreensis_:0.02366713):0.03151307):0.00409476):0.003550918):0.02935186,408_Mantidactylus_argenteus:0.09876589):0.01549161):0.01176719,((((414_Mantidactylus_lugubris:0.01093962,413_Mantidactylus_lugubris:0.01964454):0.01957266,((416_Mantidactylus_petakorona:0.02188351,418_Mantidactylus_sp_54:0.02962282):0.01039056,417_Mantidactylus_sp_53:0.01585864):0.007735665):0.02176007,(411_Mantidactylus_sp_50:0.01591603,412_Mantidactylus_sp_aff_femolugubris:0.01577987):0.03625281):0.02726847,((409_Mantidactylus_cowanii:0.02297422,410_Mantidactylus_sp_48:0.02541146):0.01565881,415_Mantidactylus_sp_49:0.01995646):0.04049033):0.0620178):0.01741951,((((379_Mantidactylus_guttulatus:0.01476012,377_Mantidactylus_sp_67:0.02595861):0.006835531,(378_Mantidactylus_radaka:0.04433376,381_Mantidactylus_sp_56:0.01603085):0.008742679):0.007657353,382_Mantidactylus_grandidieri:0.03418663):0.01455452,380_Mantidactylus_sp_55:0.02150775):0.03757507):0.01788707):0.0217953,32_Boehmantis__microtympanum:0.11526):0.02460369):0.03637452,278_Tsingymantis_antitra:0.1296843):0.01529468):0.01576727,((((((((153_Boophis__williamsi:0.06625081,((155_Boophis__microtympanum:0.01823468,154_Boophis__sp_Ca33:0.0224519):0.006192722,156_Boophis__laurenti:0.01130842):0.02655865):0.008069779,(157_Boophis__rhodoscelis:0.01205358,158_Boophis__andrangoloaka:0.01635862):0.05246241):0.02139123,((((161_Boophis__lilianae:0.08059711,(162_Boophis__baetkei:0.05304394,(163_Boophis__ulftunni:0.02063683,164_Boophis__sp_aff_ulftunni_Marojejy:0.02797494):0.02683789):0.03336085):0.01061403,(((166_Boophis__pyrrhus:0.02890992,165_Boophis_sp_CaNEW:0.008440449):0.01540894,167_Boophis__sp_aff_pyrrhus_Sahavontsira:0.008643194):0.01285027,168_Boophis__haematopus:0.05108544):0.04875392):0.005715737,(((173_Boophis__miniatus:0.07753476,174_Boophis__piperatus:0.07481207):0.008059824,(169_Boophis__picturatus:0.02291129,(170_Boophis__sp_aff_picturatus_Ranomafana:0.02382657,171_Boophis__sp_aff_picturatus_Ste_Luce:0.01364736):0.008974881):0.05298487):0.01674587,((175_Boophis__arcanus:0.07966764,176_Boophis_sp_44:0.06777548):0.01442025,172_Boophis__feonnyala:0.08721439):0.008475569):0.01886719):0.006130031,(159_Boophis__majori:0.03144675,160_Boophis__narinsi:0.03959998):0.05944029):0.005996326):0.03616055,(((((143_Boophis__rappiodes:0.02679872,145_Boophis__sp_aff_rappiodes_Betampona:0.02796246):0.01135927,144_Boophis__sp_aff_rappiodes_Ranomafana:0.04345227):0.02476408,((147_Boophis__bottae:0.01706516,148_Boophis__sp_aff_bottae_Ranomafana:0.01165973):0.01878843,146_Boophis__ankarafensis:0.03875769):0.02807976):0.009194966,((150_Boophis__erythrodactylus:0.05469501,151_Boophis__tasymena:0.03868167):0.01270141,149_Boophis_sp_CaNEW:0.07981459):0.02119564):0.0223426,152_Boophis__viridis:0.09019353):0.06184421):0.008422815,((((115_Boophis__albipunctatus:0.05710937,116_Boophis__sibilans:0.03582077):0.01374842,117_Boophis__luciae_:0.0641519):0.0407955,((118_Boophis__haingana_:0.04414829,121_Boophis__schuboeae:0.04579885):0.005213249,((120_Boophis__ankaratra_:0.02535644,119_Boophis__boppa:0.01794605):0.008083334,122_Boophis__miadana_:0.03063941):0.01646078):0.03168856):0.02265212,((((((((127_Boophis__tampoka:0.02156317,126_Boophis_sp_aff_tampoka_North:0.02177933):0.01536827,128_Boophis__sp_24:0.05687264):0.01031721,(131_Boophis__sp_aff_luteus_Andasibe:0.01759245,130_Boophis_sp_CaNEW:0.04984357):0.01725025):0.006749461,129_Boophis__luteus:0.02899735):0.0224866,125_Boophis__sp_23:0.05604285):0.008498108,132_Boophis__englaenderi:0.0621974):0.01596007,(138_Boophis__jaegeri:0.1074128,(133_Boophis__septentrionalis:0.06147083,((139_Boophis__andreonei:0.07218728,((123_Boophis_sp_CaNEW:0.02331247,140_Boophis__elenae:0.01758036):0.01957404,141_Boophis__sp_21:0.02151601):0.02474542):0.01573476,(((136_Boophis__sandrae_:0.02224789,134_Boophis__sp_36:0.006474559):0.01278904,135_Boophis__sp_37:0.01765007):0.01085397,137_Boophis__anjanaharibeensis:0.04740515):0.03492151):0.004627586):0.01514074):0.00819452):0.01387828,124_Boophis__andohahela:0.08213838):0.02046845):0.02741401):0.008378899,((((((((93_Boophis__sambirano:0.03856172,94_Boophis__sp_28:0.02829179):0.01316448,((95_Boophis__sp_Ca50:0.03956863,(96_Boophis__sp_47:0.02363059,(99_Boophis__sp_aff_mandraka_Ranomafana:0.02472501,(97_Boophis__sp_48:0.06454044,98_Boophis_sp_Ca49:0.02864866):0.009857247):0.006814728):0.01026441):0.004741854,100_Boophis__mandraka:0.01538009):0.006864835):0.02296856,92_Boophis__sp_aff_solomaso:0.05222132):0.01408736,(102_Boophis__liami:0.03407227,101_Boophis__sp_27:0.03854952):0.02684386):0.009221197,89_Boophis__sp_54:0.06146471):0.005264338,91_Boophis__sp_38:0.04771935):0.006216844,((87_Boophis__solomaso:0.03450489,88_Boophis__sp_39:0.007940936):0.01492848,90_Boophis_sp_46:0.06325028):0.02393749):0.01771322,(((106_Boophis__sp_aff_vittatus_Manongarivo:0.03932425,105_Boophis__vittatus:0.05449422):0.01601084,(103_Boophis__blommersae:0.05257497,104_Boophis__sp_aff_blommersae:0.04420578):0.04807668):0.01725539,(((((112_Boophis__marojezensis:0.0231351,111_Boophis__sp_25:0.02943578):0.01177908,113_Boophis__sp_aff_marojezensis:0.04708973):0.009152169,503_Boophis__sp_aff_marojezensis:0.01863791):0.006306644,(109_Boophis_sp_Ca52:0.01767516,(110_Boophis__sp_26:0.01573742,108_Boophis_sp_55:0.02457965):0.0119759):0.00935309):0.01262831,107_Boophis__sp_Ca51:0.04503751):0.01840417):0.02794151):0.03113674):0.009400099,(((((48_Boophis__tsilomaro:0.03916946,51_Boophis__occidentalis:0.02627666):0.01702568,49_Boophis__albilabris:0.04133629):0.01092648,50_Boophis__praedictus_:0.03603994):0.06014621,52_Boophis__masoala:0.1194272):0.008952552,(((((((((57_Boophis__quasiboehmei_:0.02641898,71_Boophis_sp_CaNEW:0.02116419):0.05734554,((73_Boophis__boehmei:0.01174276,72_Boophis__sp_aff_boehmei:0.1532447):0.01071442,(53_Boophis__sp_40:0.02506957,54_Boophis__sp_43:0.0151921):0.01206561):0.02418585):0.01082065,(55_Boophis__sp_aff_fayi:0.009013194,56_Boophis_fayi:0.0248082):0.03038419):0.01138242,68_Boophis__popi:0.0546771):0.01549783,((((80_Boophis__axelmeyeri:0.04651711,79_Boophis__sp_aff_axelmeyeri:0.04591286):0.01402008,(((83_Boophis__sp_10:0.01307444,85_Boophis__rufioculis:0.01247726):0.008409135,84_Boophis_sp_CaNEW:0.02255251):0.0568312,((76_Boophis__sp_41:0.02446077,82_Boophis__burgeri:0.03685524):0.01066691,81_Boophis_sp_CaNEW_:0.01333487):0.02756761):0.007796745):0.008917352,(77_Boophis__sp_7:0.03974659,78_Boophis_sp_CaNEW:0.03854038):0.01454424):0.006106393,(74_Boophis__sp_aff_reticulatus_Ranomafana:0.07655092,75_Boophis__reticulatus:0.0458362):0.006334795):0.01580453):0.007122221,((66_Boophis__brachychir:0.04044744,67_Boophis__entingae:0.05085877):0.01213842,((63_Boophis__sp_42:0.0275507,(64_Boophis__goudotii:0.02100248,65_Boophis__obscurus_:0.01453522):0.01389075):0.0207983,70_Boophis_sp_aff_entingae_Tsaratanana:0.02526098):0.02060787):0.01806244):0.004731522,(61_Boophis__roseipalmatus_:0.049654,62_Boophis__madagascariensis:0.04444342):0.07991417):0.008859912,(59_Boophis__spinophis_:0.004751393,60_Boophis_sp_aff_spinophis_Ambatolahy_2:0.08110375):0.05399875):0.01377265,58_Boophis__periegetes:0.0899283):0.01536329):0.008341962):0.06507022,(((35_Boophis__pauliani:0.05214026,(33_Boophis__sp_aff_pauliani_Tolagnaro:0.01731942,34_Boophis__sp_aff_pauliani:0.05655785):0.03293828):0.07503489,((((41_Boophis__idae:0.05831754,40_Boophis__sp_aff_guibei:0.04253579):0.02432309,(44_Boophis__calcaratus:0.06371831,(42_Boophis__guibei:0.02751886,43_Boophis_sp_CaNEW:0.02629049):0.01467927):0.01098873):0.02078213,45_Boophis__lichenoides:0.1014383):0.02388911,(39_Boophis__xerophilus:0.09951671,(36_Boophis__tephraeomystax:0.04202385,(38_Boophis_nauticus:0.0486687,37_Boophis__doulioti:0.0442335):0.01989769):0.05479745):0.04437096):0.01543176):0.01908609,(47_Boophis__opisthodon:0.03041842,46_Boophis__opisthodon:0.01612535):0.1290135):0.01137752):0.04900713):0.1350228);

**Supplementary References**

1. Ali, J. R. & Aitchison, J. C. Gondwana to Asia: Plate tectonics, paleogeography and the biological connectivity of the Indian sub-continent from the Middle Jurassic through latest Eocene (166–35 Ma). *Earth-Science Reviews* **88**, 145–166 (2008).

2. Ali, J. R. & Krause, D. W. Late Cretaceous bioconnections between Indo-Madagascar and Antarctica: refutation of the Gunnerus Ridge causeway hypothesis. *Journal of Biogeography* **38**, 1855–1872 (2011).

3. Dinerstein, E. *et al.* An Ecoregion-Based Approach to Protecting Half the Terrestrial Realm. *BioScience* **67**, 534–545 (2017).

4. Ganzhorn, J. U., Wilmé, L. & Mercier, J.-L. Explaining Madagascar’s biodiversity. in *Conservation and Environmental Management in Madagascar* (ed. Scales, I. R.) 17–43 (Routledge, 2014).

5. Goodman, S. M., Raherilalao, M. J. & Wohlhause, S. *The Terrestrial Protected Areas of Madagascar: Their History, Description, and Biota*. vol. I (Association Vahatra, 2019).

6. Cocca, W. Studying the processes of species diversification using the adaptive radiation of the mantellid frogs of Madagascar (Anura: Mantellidae) as a model system. (University of Porto, Faculty of Sciences, 2020).

7. Hansen, M., DeFries, R., Townshend, J. R. G. & Sohlberg, R. Global land cover classification at 1 km resolution using a decision tree classifier. *International Journal of Remote Sensing* **21**, 1331–1364 (2000).

8. Mishler, B. D. *et al.* Phylogenetic measures of biodiversity and neo- and paleo-endemism in Australian *Acacia*. *Nature Communications* **5**, 4473 (2014).
